# Axonal Velocity Distributions in Neural Field Equations

**DOI:** 10.1371/journal.pcbi.1000653

**Published:** 2010-01-29

**Authors:** Ingo Bojak, David T. J. Liley

**Affiliations:** 1 Donders Institute for Brain, Cognition and Behaviour, Centre for Neuroscience, Radboud University Nijmegen (Medical Centre), Nijmegen, The Netherlands; 2 Brain Sciences Institute (BSI), Swinburne University of Technology, Hawthorn, Victoria, Australia; Université Paris Descartes, Centre National de la Recherche Scientifique, France

## Abstract

By modelling the average activity of large neuronal populations, continuum mean field models (MFMs) have become an increasingly important theoretical tool for understanding the emergent activity of cortical tissue. In order to be computationally tractable, long-range propagation of activity in MFMs is often approximated with partial differential equations (PDEs). However, PDE approximations in current use correspond to underlying axonal velocity distributions incompatible with experimental measurements. In order to rectify this deficiency, we here introduce novel propagation PDEs that give rise to smooth unimodal distributions of axonal conduction velocities. We also argue that velocities estimated from fibre diameters in slice and from latency measurements, respectively, relate quite differently to such distributions, a significant point for any phenomenological description. Our PDEs are then successfully fit to fibre diameter data from human corpus callosum and rat subcortical white matter. This allows for the first time to simulate long-range conduction in the mammalian brain with realistic, convenient PDEs. Furthermore, the obtained results suggest that the propagation of activity in rat and human differs significantly beyond mere scaling. The dynamical consequences of our new formulation are investigated in the context of a well known neural field model. On the basis of Turing instability analyses, we conclude that pattern formation is more easily initiated using our more realistic propagator. By increasing characteristic conduction velocities, a smooth transition can occur from self-sustaining bulk oscillations to travelling waves of various wavelengths, which may influence axonal growth during development. Our analytic results are also corroborated numerically using simulations on a large spatial grid. Thus we provide here a comprehensive analysis of empirically constrained activity propagation in the context of MFMs, which will allow more realistic studies of mammalian brain activity in the future.

## Author Summary

Due to the sheer number of neurons and the complexity of their interactions, the modelling of brain activity is particularly challenging. How can computationally tractable models of brain function be developed that are nevertheless biologically plausible? The “mean field” approach, borrowed from statistical physics, is to model the average activity of populations of neurons rather than the behaviour of individual neurons. While a large number of promising theories have been developed with this approach, they fall short of biological fidelity in the way interactions between distant populations have been modelled. In particular, it is often assumed that all neurons interact via connections of very similar conduction velocity, when in fact experiment suggests quite the opposite: populations of neurons are connected by axonal fibres with a broad range of velocities. We develop here activity propagators that provide for the first time the ability to realistically and efficiently simulate connectivity in mean field theories, and demonstrate how to use them to fit successfully experimental data from both human and rat. With our novel propagators, one can thus study on an empirical basis the role of activity propagation in both healthy and diseased mammalian brains.

## Introduction

Since the introduction of continuum formulations for the dynamics of neural masses in cortical tissue [1]–[6], the interest in this class of neural mean field models (MFMs) has been steadily growing. MFMs have been used to describe a wide range of phenomena by acting as a mesoscopic bridge between the results of neuroimaging and the underlying anatomy, physiology and pharmacology. The growing list includes: the effects of anaesthetics, tranquillizers, and stimulants [7]–[10], gamma band oscillations [11]–[13], epilepsy [14]–[18], sleep [19],[20], and evoked potentials [21],[22]. A recent review by Deco et al. [23] details both the theoretical framework and some general principles for the application of such theories.

However, MFMs face severe technical difficulties when dealing with non-local neural activity, which is propagated across cortex by long-range axonal fibres. In order to incorporate the effects of such distributed activity a number of assumptions are typically made, the most important being a single value for the activity propagation delay between distant neural masses. This is the case even in otherwise sophisticated models, for example in those combining MFMs with Dynamic Causal Modelling (DCM) [24]. Most modelling approaches (e.g., [25],[26]) follow here the lead of the seminal paper by Jirsa and Haken [27], who employed several simplifying assumptions to describe long-range activity propagation with a partial differential equation (PDE). However, their ansatz still assumes a single value for the cortico-cortical axonal conduction velocity, and thus conduction delays between neural masses are exactly proportional to their distance with one uniform constant. We will show below that approximations made in deriving the actual propagation PDE result in an implicit velocity distribution, which nevertheless due to its origin remains strongly peaked at maximum conduction velocity and is one-sided, i.e., there is an infinitely sharp cut-off at maximum speed. MFMs typically describe neural masses consisting of 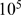 to 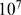 neurons each. Thus even if the conduction velocity of one axon can be approximated well with a single conduction velocity, one should expect a distribution of conduction velocities between neural masses given the many axons involved. Empirical measurements of conduction velocities, either directly via conduction latencies or indirectly via fibre diameters, indeed suggest that conduction delays are rather broadly distributed. Initial attempts by Hutt and Atay [28],[29] to incorporate broad axonal velocity distributions in a particular, spatially continuous MFM have revealed that such broad distributions maximize the speed of travelling front solutions. This may indicate the influence of natural selection optimizing information transmission in cortex.

Hutt and Atay [28],[29] made use of a general integro-differential formula for activity propagation, which allows a straightforward introduction of velocity distributions. It is just this integro-differential formula, which is commonly simplified towards a PDE [27]. As discussed for example by Liley *et al.* [26], local PDE formulations offer a number of significant advantages over their non-local (integral) counterparts. In particular, they enable the use of powerful analytical and numerical analysis methods, at least for specific spatial wavenumbers, and allow the application of standard numerical techniques for the solution of MFMs. The latter point is particularly important for large-scale simulations, see for example [9],[13], where computation speed is essential. As derived in [30] by the present authors, one can always extract the velocity distribution implied by the PDE formulation of an MFM. But so far the exact form of these distributions have been largely an accidental side product of approximations. It is hence no surprise that the velocity distributions of models in current use are unsatisfactory. Incorporating a sensible velocity distribution into an analytically and numerically tractable PDE formulation has not been achieved before.

Motivated by physiological and anatomical fidelity on one hand, and by computational necessity on the other, we here introduce a novel PDE formulation describing the propagation of cortico-cortical axonal activity that incorporates monotonically decaying synaptic connectivity with a smooth unimodal distribution of axonal conduction velocities. We obtain good fits with our new model to experimental data on conduction velocities derived from myelinated fibre diameter measurements in the human corpus callosum [31]. This allows for the first time to simulate long-range conduction in humans based directly on experimental findings. A straightforward extension of initial propagator ansatz also allows us to fit data from lower mammals, which generally feature less small diameter (myelinated) fibres. Studying activity conduction in animal cortex is important in its own right, but also significant for the suitability of animal models for human studies. For example, the CoCoMac database [32],[33] contains precise information on the connectivity of macaque cortex from extensive tracer studies, which cannot be obtained similarly from humans since such techniques are lethal. While CoCoMac connectivity can be mapped to human cortex [34] and calibrated with human connectivity data from non-invasive Magnetic Resonance Imaging [35], the question would remain whether similar anatomical connections actually serve the same function. Clearly an improved understanding of the dynamics of activity conduction in animals and humans is of great significance to this question.

We obtain reasonable fits with our extended ansatz to extensive unmyelinated and myelinated data from rat subcortical white matter [36], and discuss briefly the clear differences that exist to the fit to human callosal data. Finally, we also analyse analytically and numerically the dynamical impact of using our new propagator. Following the methods in Coombes et al. [30], we can show that in contrast to the most commonly used long-wavelength propagator, our realistic velocity distributions enable the formation of spatio-temporal patterns for smaller perturbations in mean neuronal firing rates. This may follow more closely the biological situation, where a range of energetic constraints need to be negotiated in order to ensure that pattern formation, and thus perception, occurs in metabolically optimal circumstances. We confirm these results with some explorative computational simulations on large spatial grids using our novel propagator. So far, conduction parameters in mean field models have been either chosen largely arbitrarily from a wide range of plausible values, or adjusted freely to help reproducing the phenomena under investigation. Our fits to human and rat data, and future fits to other experimental data using our methods, constrain propagation parameters empirically and independently. This will reduce considerably the uncertainties of future predictions using the mean field framework.

## Model

### Dispersive propagator

In most neural field models developed to date the activity variables that are spatially propagated are the local mean neuronal population firing rates, 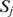. Because action potentials propagate with a finite conduction velocity, the mean rate of arrival of pre-synaptic impulses 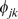 to cells of type 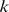 from neurons of type 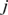 can be written as a time-retarded integral of the respective distant local mean excitatory neuronal firing rates:
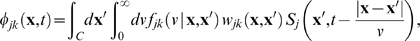
(1)

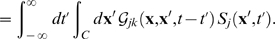
(2)where spatial integration occurs over a two-dimensional planar cortical sheet 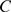 (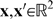). The distance-dependent velocity distribution function 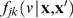 takes into account that fibre paths with different conduction velocities can exist between different domains. This conditional distribution is normalised such that 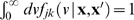. The function 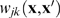 is the synaptic footprint that describes the geometry of network connections. The distance dependent Green's function, 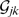, is defined as:
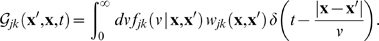
(3)


In the absence of detailed anatomical data it is common practice to consider synaptic connectivity functions to be homogeneous and isotropic so that 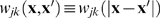. We will also assume that this restriction applies to the velocity distribution functions, i.e., 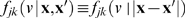, and therefore 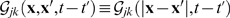. This assumption of isotropy can be relaxed at the price of increased computational effort [30],[37],[38], as will be discussed below in a separate subsection. The right hand side of (2) now has a convolution structure, and its Fourier transform, 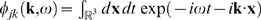 yields
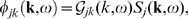
(4)where 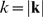. If 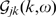 has the form 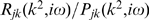 then the integro-differential Eq. (2) can be written as the equivalent PDE 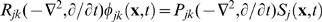, i.e., the corresponding partial differential operators are obtained with the Fourier replacements 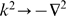 and 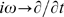.

The most common propagator form used in mean field models of electroencephalographic activity derives from the following simple ansatz for the Green's function: an exponential decay with distance of propagated firing rates is combined with isotropic conduction
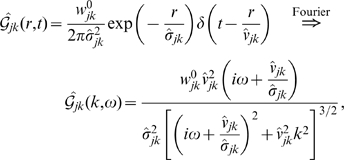
(5)where 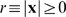 and axonal velocity 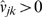 together imply the *causal* conduction of activity through a Dirac 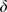 distribution of delays. The normalization constant 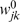 counts the total number of synaptic connections made by the axonal fibres originating from neurons of type 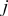 that terminate on neurons of type 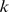. The exponential decay with the characteristic distance scale 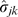 should be understood as due to diminishing connectivity [39], rather than as decay of the amplitudes of the action potentials themselves. The Fourier domain propagator in Eq. (5) is non-polynomial, but can be approximated for small 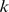, and hence long wavelengths 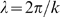, with a polynomial form. Setting 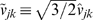 and 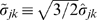 we obtain
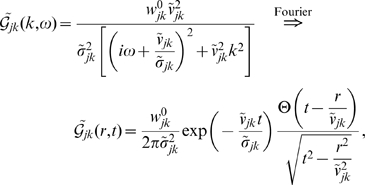
(6)where 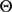 is the Heaviside step function, which now maintains causality. We will subsequently refer to this as the *long-wavelength* approximation. The standard inhomogeneous, 2-dim. telegraph equation [25]–[27] results
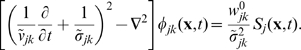
(7)


Note that (7) is a special case. If we substitute
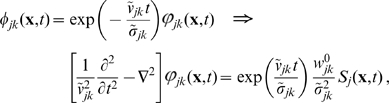
(8)then 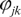 obeys an inhomogeneous wave equation. Note that Eq. (8) corrects a sign error in Eq. (61) of Ref. [25]. The approximate impulse response 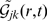 in Eq. (6) can hence be recognized as that of a 2-dim. wave with velocity 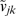 multiplied by an exponential decay with *velocity-dependent* distance 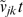.

The infinitely precise conduction delay 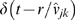 of ansatz Eq. (5) is at odds with the broadly distributed delays measured by experiment. In the next section we will show that the long-wavelength approximation largely inherits this problem. An obvious amelioration would be to use a Gaussian normal distribution of delays:
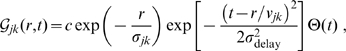
(9)where 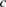 is an appropriate normalization constant and the Heaviside 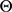 enforces causality. However, Eq. (9) leads to the same type of *non-polynomial* Fourier structure as Eq. (5), only multiplied with 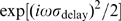. Thus again an approximation would be needed to obtain a polynomial form and hence a PDE. A key observation is that the problematic fractional power 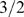 arises from the spatial Fourier transform of 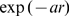 terms, where the 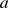 are independent of distance but can depend on time, and that we can eliminate all such terms from the ansatz by setting 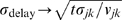:
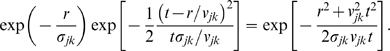
(10)


We can Fourier transform this expression, first spatially (which is equivalent to a zeroth order Hankel transform) and then temporally, even if it is multiplied with powers of 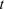. Hence we now propose the following Green's function:
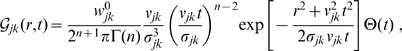
(11)where 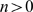 and 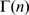 is the Gamma function with 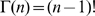 for integer 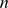. The corresponding Fourier domain propagator is
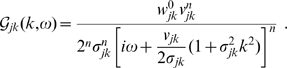
(12)


Using this to propagate local mean firing rates according to Eq. (4) is hence equivalent to the following two-dimensional PDE
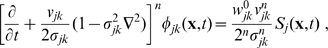
(13)where only 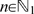 will realize any practical benefits for analysis and computation. Note that for 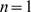 this corresponds to a two-dimensional, inhomogeneous cable equation. We will subsequently refer to this novel ansatz as the *dispersive propagator*.

It should be emphasized at this point that single propagation PDEs, like the dispersive Eq. (13) and the long-wavelength Eq. (7), imply that firing rate activity passes continuously between any two arbitrarily chosen cortical locations. However, cortico-cortical fibres are known to also selectively connect separated areas of cortex in a direct manner, see for example Ref. [40]. Such non-local propagation cannot be modelled with the PDE descriptions of activity conduction described so far. To include non-local effects one must either resort again to the general integral equations, or map cortex to a mixture of overlapping patches based on a chosen PDE description. Recently good progress has been achieved for the latter option [38], in particular also by turning such descriptions into a kind of DCM [41], which makes possible robust fits to experimental neuroimaging data. Our efforts here are complementary to these pioneering works, since we are concerned with obtaining physiological conduction velocity distributions in the typical PDE framework. For example, the long-wavelength approximation Eq. (5) in Ref. [38] could be replaced with our dispersive Eq. (13) as basis for considering non-local effects, thereby increasing the realism of the non-local conduction model even further. We will explain in a separate subsection below in what way anisotropy and inhomogeneity can also affect the extraction of velocity distributions from experimental data.

In the original ansatz of Eq. (5), impulses would arrive at distance 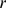 from a source precisely after a time 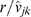 had passed. The extension in Eq. (9) was constructed such that the impulses would arrive with a Gaussian normal distribution of delays having mean 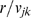 and standard deviation 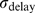. We can recover this from the respective Green's functions by computing the statistical characteristics of delays, appropriately normed by the decay of connectivity to distance 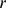:
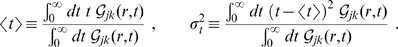
(14)


Thus indeed 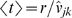 and 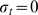 for the original ansatz Eq. (5), but for the long-wavelength approximation Eq. (6) thereof one finds instead
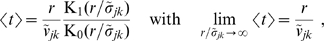
(15)

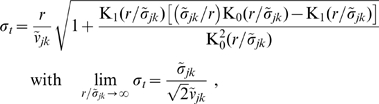
(16)where 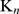 is the 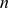
th order modified Bessel function of the second kind. Similarly for the Gaussian extension Eq. (9) we obtain the expected results 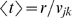 and 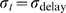, but for our new dispersive propagator we find instead
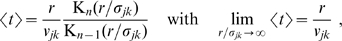
(17)

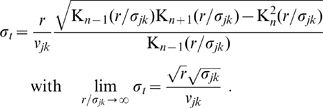
(18)


From the results for 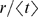 one can see that the characteristic long-wavelength (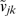) and dispersive (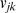) velocities still indicate the axonal conduction velocities, but only on average and at *large* distances. A “large” distance means here one much greater than the characteristic decay scales of connectivity, 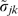 and 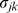, respectively. At large distances the standard deviation of delays 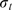 becomes constant for the long-wavelength approximation, but 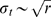 for the dispersive propagator, i.e., it *grows* with the square root of distance. We also see that 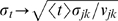 at large distances, which recovers the substitution of 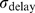 leading to Eq. (10). Note finally that long-wavelength 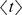 is identical to the dispersive 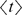 at all distances for 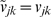 and 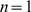.

### Synaptic connectivity and velocity distribution

By integrating the dispersive Green's function Eq. (11) over time we obtain the implied dependency of synaptic connectivity with distance
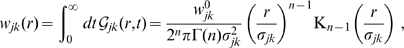
(19)

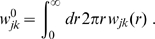
(20)


Here 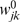 counts the total number of synapses formed and 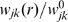 is the probability distribution of the synaptic footprint, i.e., the likelihood that a synapse forms at distance 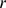, where 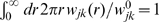. Connectivity 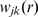 remains finite for 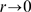 only if 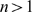, in which case 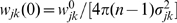. In practice the 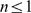 divergence for 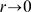 is of little concern, as neural field models are not meaningful below some minimal size 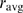 over which mean population activity is defined. The contributions of synaptic connections within the disc 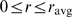 to the total number of synaptic connections 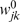 vanishes for 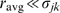 for all 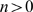. Eq. (19) should be compared with the connectivity function for the long-wavelength approximation Eq. (6)
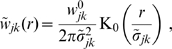
(21)with 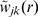 normed to 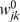 as in Eq. (20). We note again an equivalence to Eq. (19) with 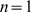.

Both the dispersive and the long-wavelength propagator thus have synaptic footprints decaying with distance 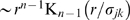, where for the latter 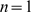. However, experimental counts of synaptic connectivity usually have been fit with the simpler exponential decay
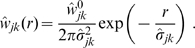
(22)


Thus the question arises whether dispersive connectivity is compatible with data that apparently fit an exponential decay, and whether one can use such previous fit results to constrain also the dispersive propagator. An exponential decay is also what the original ansatz Eq. (5) used. Hence in previous works it has been assumed that model and fit scale are basically the same quantity. But it will become clear now that after the long-wavelength approximation Eq. (6) this is not correct anymore. Let us assume that the dispersive synaptic footprint Eq. (19) with parameters 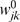 and 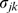 represents the true underlying distribution of connectivity, and that from it parameters 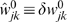 and 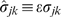 are estimated with a fit assuming the exponential distribution Eq. (22). Therefore we wish to determine which 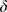 and 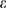 best corrects for the mismatch. In practice, experimental counts of synaptic connections are usually sorted into distance bins 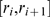, where 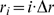 with 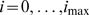. We can scale 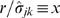 and 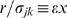, where 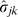 is known from the experimental fit. The counts per bin are then
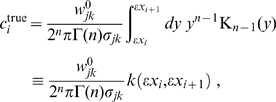
(23)

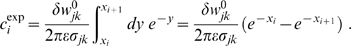
(24)


A usual least square fit of 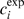 to 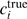 will hence implicitly minimize
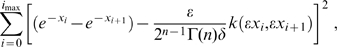
(25)and we can minimize this expression explicitly to determine 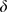 and 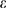. To give a numerical example: assume 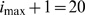 bins of width 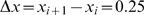, i.e., the bin size was a fourth of the fitted 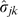 and in the last bin connectivity had decayed to less than one percent of maximum. For different powers 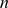 we can then obtain numerically scaling factors 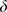 and 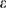:
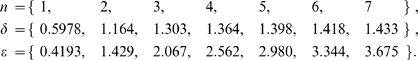
(26)


**Figure 1:**
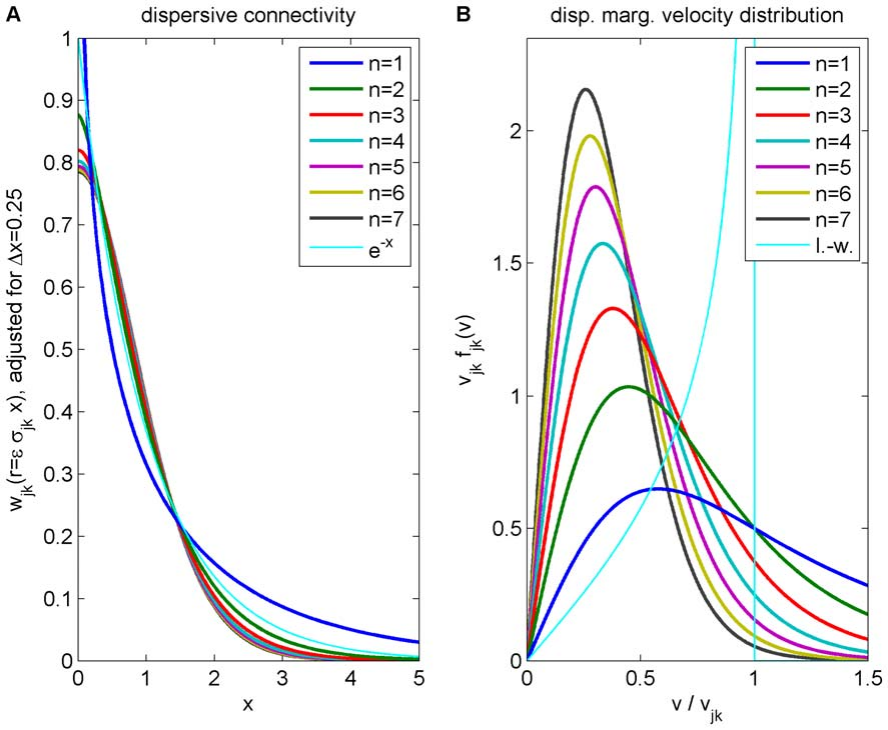
Dispersive propagator: synaptic connectivity and marginal velocity distribution. (A) Synaptic connectivity 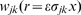 for different powers 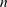, which has been adjusted to match an exponential decay (thin curve). While the curves are continuous here, adjustment with Eq. (25) assumes a bin size 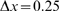, see text for details. (B) Marginal velocity distribution 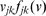 for different powers 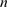. Note that concerning the dimensionless ratio 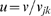 one obtains 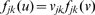. The long-wavelength approximation 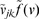 of Eq. (36) is shown for comparison as thin curve. See Eqns. (19) and (32) for (A) and (B), respectively.

We find that the normalization correction 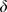 has an asymptotic value for large powers 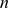, whereas the decay correction 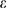 grows as 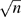. The resulting synaptic connectivity is shown in Fig. 1A. For simplicity we have assumed here that 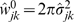, i.e., that 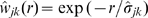. The dispersive curves are hence 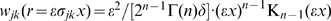 with the scaling factors derived above. While we show continuous curves here, the correction was performed for binned data. It is obvious from the reasonably close match that dispersive connectivity may well be mistaken for an exponential decay, given the large statistical and systematic errors typically involved in synaptic counts. Note that the 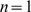 divergence for small distances would not be visible in a binned count. Nevertheless, it is obvious that the 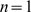 case, and hence the long-wavelength approximation, does not match an exponential decay better than higher powers of 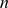. Furthermore, for 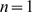 in this example we find the optimal scaling 
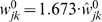
 and 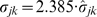. In general for long-wavelength models one should actually choose 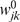 and 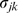 which are significantly larger than those measured in experiments. Note that our long-wavelength decay scale absorbed an expansion factor 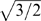 to keep Eq. (6) simple. Without this, scaling by 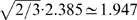 would be required here.

Eq. (3) enables us to determine the underlying conduction velocity distribution of the axonal fibres that arises from our newly proposed dispersive propagator. Thus we obtain
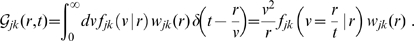
(27)


Using the Green's function Eq. (11), the distance-dependent velocity distribution 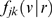 becomes
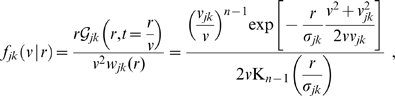
(28)which has a maximum at
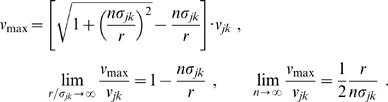
(29)


**Figure 2:**
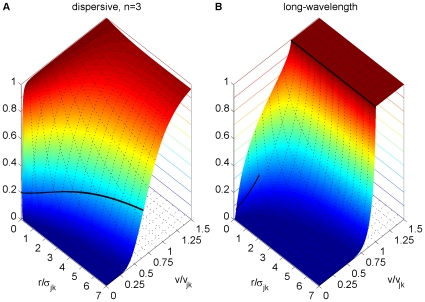
Cumulative distance-dependent velocity distributions: dispersive propagator vs. long-wavelength approximation. Shown are cumulative distributions integrated over 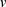 as in Eq. (30). Dotted black lines on the base and on the plot surface show a grid of 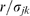 and 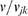 values, solid black lines on the plot surface show the positions of the maxima of the unintegrated distributions. (A) Dispersive propagator for 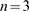, where 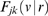 corresponds to Eqn. (28). (B) Long-wavelength approximation, where 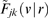 integrates Eqn. (33). We set 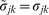 and 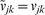 for comparison.

The 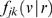 distribution indicates the probability of conduction velocity 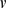 at a given distance 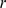. As far as experimental data are concerned, this distribution is appropriate for measurements of conduction latencies between brain regions. For that case we can consider 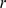 to be fixed and note that 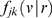 is properly normed as a *conditional* probability distribution in 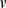, i.e., 
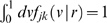
. The time 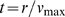 indicates the moment when most propagated activity arrives at once in a region. One can speculate that this has the highest likelihood to induce a signal visible over local background activity. According to the first limit in Eq. (29), we then expect latency data for distant (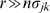) regions to measure conduction velocities 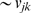. Fig. 2A shows a plot of the cumulative distribution
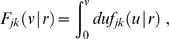
(30)corresponding to Eq. (28). We prefer to show the cumulative distribution here, because of the large variations of 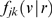 in the shown range of 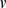 and 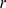. Furthermore, this allows a direct comparison with the long-wavelength approximation later on. The sigmoidal shape of 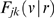 in 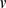 corresponds to the unimodal form of 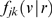. The position of the mode 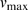 of 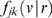 is indicated by a solid black line on the 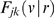 surface. That 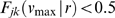 indicates that the distribution is skewed towards higher velocities. However, we can see that the distribution becomes less skewed for larger 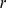. Furthermore, we see that neuronal populations at greater distances on the cortical surface are connected by faster fibres. While from a functional perspective this makes intuitive sense, there is at present no direct anatomical or histological evidence for this. We discuss some indirect evidence below. The second limit in Eq. (29) shows that higher order 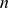 distributions describe overall slower connectivity for the same 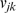.

The distance-dependent connectivity function for each fibre system of velocity 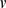, 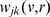, is then
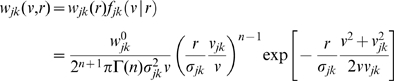
(31)where 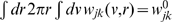, and 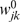 counts the total number of synapses formed. Hence, 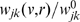 defines the *joint* probability distribution for propagation with speed 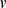 to distance 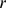. The *marginal* propagation velocity distribution over all 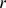 is then
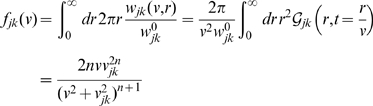
(32)where 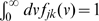. As far as experimental data are concerned, this distribution is appropriate for measurements of local fibre diameter statistics, which can be related to conduction velocities. Such statistics catalogue all fibres passing through a local slice, irrespective of the distance between the neural populations they connect. This corresponds to integrating out the distance in Eq. (32).

We show the marginal velocity distribution (multiplied by the constant 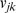) in Fig. 1B for several different powers 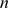. The rapid sharpening up of the distribution for higher powers is readily apparent. The statistical characteristics of the dispersive 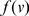 distribution are collected in Tab. 1; note also that it becomes a beta-prime distribution with 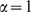 and 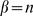 under nonlinear scaling 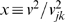. For 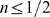 both the mean and standard deviation of the dispersive 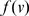 do not exist, like for a Cauchy random variable, and for 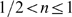 the mean exists but not the standard deviation, due to the tail-thickness of the distribution. Thus at 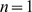 large variations of the conduction velocity are probable. The coefficient of variation 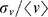 asymptotes to 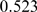, even then indicating a broad distribution. For 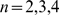 the corresponding velocity distributions already have 66%, 79% and 84%, respectively, of this maximal “sharpness”. Skew 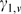 exists for 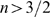 and indicates preference for higher velocities. The mode 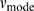 of the marginal dispersive velocity distribution is smaller than 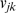, see Tab. 1. This is more pronounced for higher order 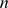 due to a larger fraction of slower fibres. By contrast, the mode 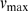 of the conditional dispersive velocity distribution approaches 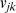 for large distances, see Eq. (29), but again more slowly for larger 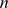. Both mode speeds are identical in the dispersive case for 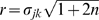, where below this distance 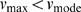 and above this distance 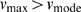. As we see from this example, comparisons of the dominant speeds – 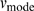 estimated from fibre diameters in a local slice and 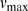 from latencies between distant brain regions – can be used as an experimental probe of the underlying connectivity. For fibre distributions like the dispersive one, in which more distant regions are connected by faster fibres, one would expect distance-dependent relations between 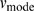 and 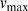 qualitatively similar to the ones just described. Latencies observed at different distances could complement the experimental constraints from local fibre diameter measurements quantitatively, too. However, the difference between 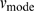 and 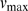 becomes more significant for measurements at larger distances, where unfortunately one would also generally expect worse signal to noise ratios. Thus it is currently unclear whether such comparisons are in fact feasible experimentally beyond a qualitative consistency check. Nevertheless, there is a chance to gain significant new insights into brain connectivity here using comparatively “simple” techniques, or even from a re-analysis of previously obtained data.

**Table 1:** Statistical characteristics of the dispersive, long-wavelength, and difference marginal velocity distributions.

	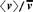	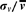	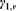	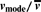	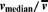
dispersive 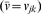	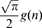	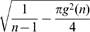	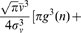 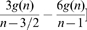	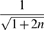	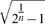
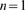	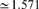	∄	∄	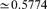	1
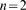	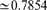	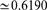	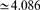	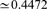	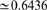
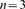	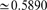	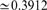	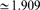	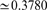	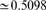
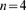	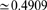	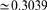	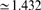	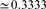	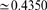
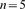	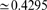	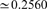	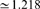	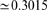	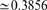
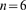	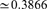	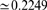	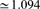	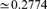	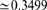
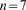	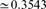	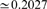	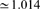	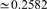	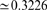
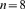	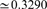	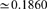	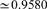	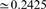	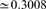
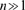	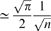	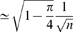	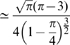	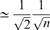	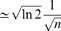
	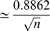	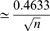	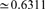	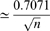	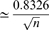
long-wave. 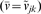	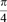 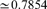	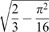 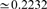	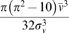 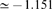	1	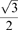 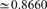
difference 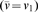	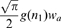	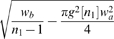	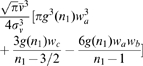	numerical	numerical
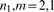	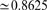	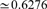	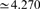	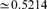	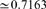
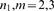	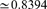	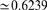	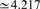	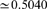	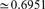
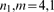	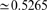	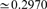	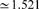	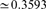	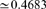
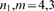	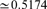	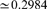	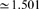	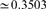	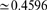
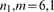	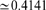	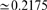	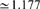	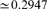	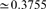
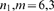	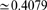	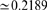	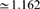	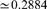	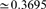
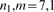	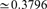	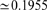	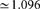	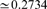	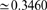
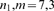	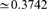	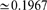	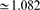	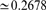	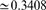
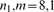	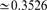	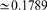	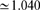	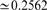	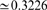
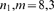	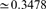	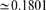	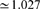	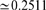	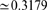

Statistics are shown for the following marginal velocity distributions: dispersive Eq. (32), long-wavelength Eq. (36), and difference Eq. (51). The characteristic velocities 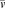 for these three distributions are 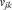, 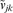, and 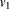, respectively. 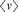, 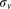, and 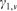 are the mean, standard deviation, and skewness in 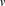, respectively. In order to achieve a compact notation, we have defined 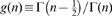, where 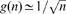 for 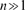. Further, we use 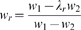 with 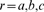 and 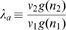, 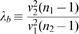, and 
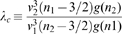
. For 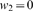 one finds 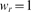, as the difference propagator turns into the dispersive one. We have not found a closed analytic form for the mode 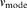 and median 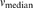 of the difference distribution, but they can be computed numerically. Further definitions needed for the evaluation of the difference distribution statistics are collected in Eqns. (43) and (52).

The distance-dependent velocity distribution for the long-wavelength approximation Eq. (6), unlike for the dispersive propagator, is truncated for velocities greater than 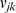:
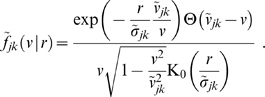
(33)


Again for 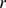 fixed 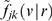 becomes a conditional probability distribution in 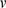 appropriate for comparisons with experimental conduction latencies. Fig. 2B shows a plot of the corresponding cumulative distribution 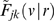, integrated as in Eq. (30). Note that 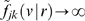 for 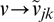, whereas 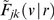 is well-behaved in the limit and hence can be plotted easily. For 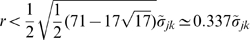, there is a local maximum of the distribution at small velocities:
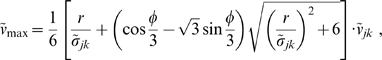
(34)

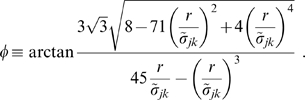
(35)


For very small 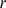 this maximum even formally becomes dominant, but at such distances the MFM loses validity. Thus the global maximum is in practice always determined by the cut-off 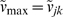. The position of the maxima of 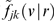 is indicated in Fig. 2B by two solid black lines on the surface of 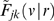. The corresponding marginal velocity distribution, which can be related to measurements of axonal diameters, is given by
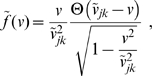
(36)and its statistical characteristics also are collected in Tab. 1. We see that this distribution is very sharp, with a coefficient of variation 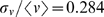, and skewed to lower velocities. Indeed, high velocities are cut off at 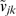. Note that the mode of the marginal distribution is the same 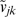 as the maximum velocity between distant brain regions of the distance-dependent distribution. Thus here we would predict that fibre diameter and latency measurements derive roughly the same conduction velocity. Basically the long-wavelength approximation retains the original sharply peaked velocity distribution of fibres with a single conduction velocity 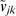. If the comparison between conduction velocities derived from diameter measurements and latencies can achieve sufficient statistical significance, then this would allow an experimental distinction between the dispersive and long-wavelength propagators. We consider investigating inter-hemispheric connectivity between contra-lateral brain regions as promising, because it is heavily dominated by just one fibre type (myelinated fibres), with fairly homogeneous regional expression across large distances. This adds particular significance to our fit of diameter data of myelinated axons from human corpus callosum performed below.

#### Incorporating anisotropy and inhomogeneity

In our presentation of the dispersive propagator, and the subsequent derivation of the conditional and marginal velocity distributions, we have assumed both isotropy and homogeneity of the corresponding connectivities. It is fortunate that these restrictions can be relaxed, given that neither homogeneity nor isotropy would be expected to hold fully in real brains, particularly not so for long-range connectivity. First, inhomogeneities will be described well by our equations in an average sense, as long as they are relatively small and random according to some unimodal distribution, e.g., a normal distribution. This fits well with the general MFM approach of describing only the “mean fields” of cortex. Further, the parameters may vary in an arbitrary inhomogeneous fashion over distances farther away than a few times the characteristic scale of synaptic connectivity 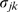, without causing local complications. Conducted over these distances a local pulse will have mostly decayed away, hence the PDEs remain valid. This suggests a separation of cortex into regions of “homogeneous enough” conduction properties. If the inhomogeneous variation of conduction properties across cortex is nevertheless smooth, then even a single PDE with matching spatial variation of parameters could be used as model. Otherwise one would have to take special care at the boundaries.

Second, to describe anisotropic conduction a generalization to “patchy” propagators is possible. Work by Robinson [37] has shown that one can generate basically arbitrary angular modifications of conduction properties at the price of introducing more PDEs. Basically this technique relies on a spatial Fourier decomposition of long range connectivity. Hence the sharper the anisotropy one wishes to describe, the more PDEs one has to employ. See for example Ref. [30], where sinusoidal variations in two principal directions required the solution of four coupled complex PDEs, instead of one real PDE. In practice a compromise between biological fidelity and numerical complexity has to be made. Consider then the following “patchy” Green's function
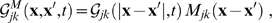
(37)which is homogeneous but anisotropic. It allows the specification of anisotropic connectivity through a decomposition into an isotropic Green's function 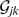 and an anisotropic, but time-independent, modifier 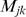. Now we can use Eq. (3) for 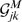 and integrate over the Dirac 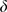-distribution, as for Eqns. (27) and (28), but without any assumption of isotropy. The synaptic footprint is again the integration over time of 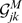, like in Eq. (19). Thus the conditional velocity distribution becomes here
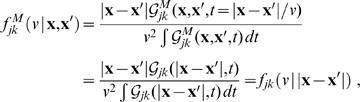
(38)i.e., the anisotropic modifier 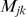 cancels out and the conditional velocity distribution 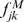 is found to be isotropic, and identical with the 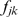 of the isotropic Green's function 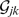. Thus an isotropic conditional velocity distribution is entirely compatible with anisotropic connectivity.

Rewriting Eq. (3) in polar coordinates, 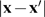 and 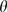, one finds that in general
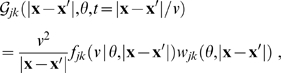
(39)and thus the potential anisotropy of propagation velocities is independent of any evidence or assumptions regarding the anisotropy of synaptic connectivity. In other words, how fast the fibres connecting two regions are is a different question to the number of fibres that connect these two regions. Hence even for real brains one can start with the parsimonious isotropic assumption for the conditional velocity distribution 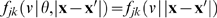, and assume that anisotropies are due only to 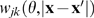. Then the fibre system is potentially anisotropic, but where fibres grow their distribution of conduction velocities is not dependent on the direction in which they are growing. Further, define the “angular average”
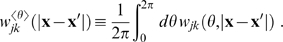
(40)


Then the generalization of Eq. (32), which assumes that the conditional velocity distribution is isotropic but allows for anisotropy in the connectivity, can be written as
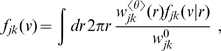
(41)

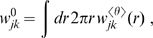
(42)where we have set 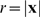 again. This clearly depends only on 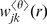, and may very well be practically indistinguishable from isotropic conditions. For example, a fibre system with one strongly dominant direction 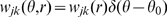, which is roughly the case within corpus callosum, yields the same isotropic 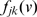 through the renormalization of 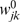. For these reasons we will continue with the assumption of isotropy for fits of the marginal velocity distributions to data. However, more precise data on both connectivity and conduction latencies may well make possible in future to disentangle anisotropies further, potentially showing that our parsimonious assumption of an isotropic conditional velocity distribution was incorrect. One also needs to keep in mind that for simulations of cortex the introduction of inhomogeneous regions and “patchy” propagators will likely be required to achieve good biological fidelity, even if one assumes isotropic velocity distributions. In this regard the methods of Daunizeau *et al.* [38] may prove particularly useful, which systematically map conduction PDEs to heterogeneous cortico-cortical connectivity in the human brain.

### Difference propagator

Finally, there appears to be a general trend in experimental data that higher mammals have a larger proportion of small diameter fibres, see for example the discussion in the section “Species differences” of [31]. We will encounter this phenomenon when trying to fit human [31] and rat data [36]. Small fibre diameters correspond to low conduction velocities, as we will see in detail below. Unfortunately the dispersive propagator predicts too much low velocity conduction, and thus a too large fraction of small diameter fibres, to fit the rat data well. Whereas the long-wavelength approximation fails entirely to describe either human or rat data, but because of high, not low, velocity conduction: its marginal velocity distribution is sharply peaked close to an upper velocity limit, while all data require a broad, unimodal velocity distribution. We have been unable to find another *single* propagator equation, which both yields the polynomial Fourier structure leading to a PDE and describes the data from lower mammals better.

A constructive approach for dealing with this problem posed by animal data has however proven successful. The basic idea is to subtract two dispersive propagators 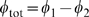, where the second dispersive propagator conducts activity more slowly, so that the resulting distribution is reduced at small velocities. This construction we will then call the *difference propagator*. Before we provide further mathematical details, we wish to justify this method with regards to the actual biology it is supposed to describe. Clearly there are no “anti-fibres” in the brain, hence 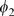 and therefore also 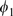 lack any direct biological meaning taken separately. But the biological meaning of the constructive solution 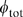 is not necessarily compromised, since in the end it is actually 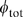 which is compared with empirical measurements. The dispersive and long-wavelength propagators we have investigated so far are biologically meaningful and appropriate because of the following characteristics: First, they correspond to a Green's function non-negative for all positive times and distances. This implies that a positive local pulse also leads to positive pulses arriving at distant synaptic terminals. The impact of these pulses may be “negative”, if they excite inhibitory populations, but the action potentials themselves do not somehow change sign. Second, synaptic connectivity has a roughly exponential decay with distance, as is appropriate for describing background connectivity in the brain. Third, the distance-dependent velocity distribution has a dominant mode, i.e., there is a preferred conduction velocity leading to typical latencies between brain regions. Fourth, the marginal velocity distribution has a shape which compares favourably with fibre diameter distributions. We will construct our difference propagator so that it shows all these characteristics. Hence while it may be less intuitive, and requires more computational effort, 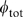 will be as valid in terms of biology as the dispersive and long-wavelength propagators.

We first compute the ratio of two dispersive Green's functions 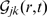 from Eq. (11), which have different parameters
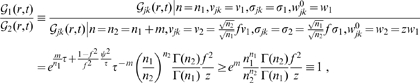
(43)with normed spatial variables 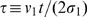 and 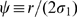. The inequality is valid for powers 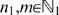, and thus 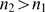, as well as factor 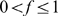, and we have set
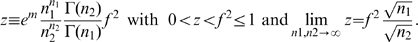
(44)


If we now define
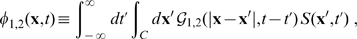
(45)

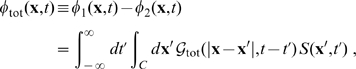
(46)then it is clear that for 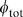 local firing 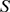 will be propagated with a combined Green's function 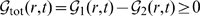. By construction we have made certain that no unbiological “negative pulses” can arise here in spite of the subtraction. Thanks to the linear combination, the distributions are computed trivially, e.g., synaptic connectivity is
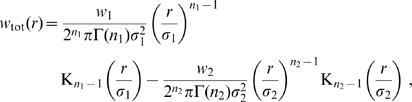
(47)

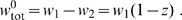
(48)


Note that as integral over 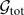, see Eq. (19), 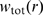 and hence 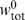 must be positive, since 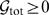 and not zero in the entire integration range. In practice 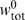 is the biological quantity and determines 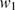 via Eqns. (48) and (44). We can again compute how this connectivity compares to an assumed exponential decay, as explained at Eq. (25). The sum to be minimized becomes now
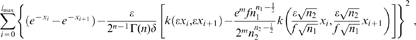
(49)where 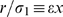. For different powers 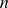 we obtain here scaling factors 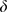 and 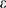 which are similar to those of the dispersive propagator:
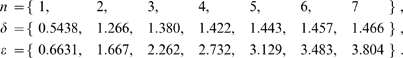
(50)


**Figure 3:**
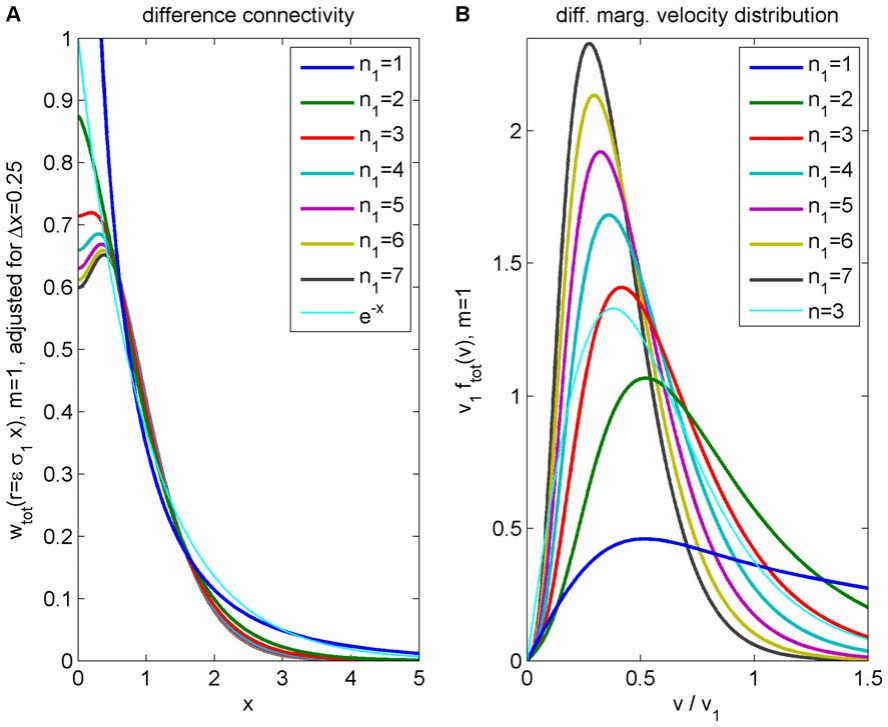
Difference propagator: synaptic connectivity and marginal velocity distribution. This figure is like Fig. 1, but for the difference propagator with 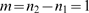. (A) Synaptic connectivity fit to an exponential decay (thin curve), Eqns. (47) and (49) are used. (B) Marginal velocity distribution Eq. (51). The dispersive 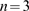 case is shown as thin curve for comparison.

In Fig. 3A the corresponding difference connectivity is shown. We see that it may become feasible to measure experimentally the deviation to an exponential decay in particular for small 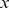 and high powers 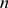, though overall the shape is still roughly exponential. The distance-dependent velocity distribution 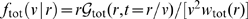 and the distance-dependent connectivity 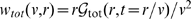 are of course also positive. It is straightforward to show that for 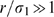 the conditional distribution 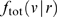 is indeed unimodal, with the maximum given by Eq. (29) upon replacing 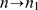, 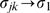, and 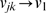. At 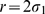 the mode velocities of the dispersive and difference propagators already differ by less than 10% for 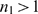.

Finally we can compute the marginal velocity distribution
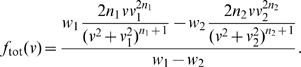
(51)


Its statistical characteristics can again be found in Tab. 1. As before mean 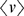 only exists for 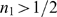, standard deviation 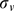 only for 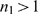, and skewness 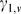 only for 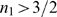. No further condition is required, since 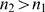. We have not been able to find analytic expressions for 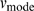 and 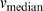 for unspecified powers 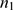 and 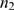. However, computing them numerically for chosen powers is straightforward. Since we wish to deplete 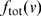 at small 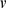, we want to maximize positive skewness 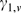 using the still available factor 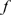. There is a clear mode of 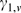 in the range 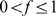, but again it is too difficult to find it analytically. Instead we obtain 225 numerical solutions for 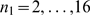 and 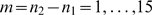, and then obtain a good three parameter fit for maximum skewness:
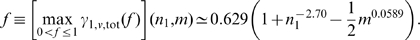
(52)


With Eq. (52) we complete the specification of our difference propagator. In practice then, the difference propagator can be computed using two PDEs
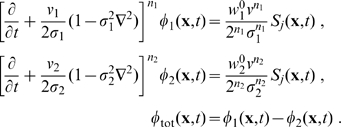
(53)where only four parameters are actually free: 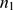, 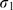, 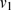, and 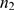. All the other parameters are dependent, see Eqns. (43), (44) and (52). Furthermore, 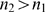 is also required. Thus in comparison to the dispersive propagator only one additional parameter is introduced here: the chosen power 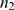 of the subtracted dispersive propagator. While the variables 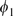 and 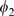 have no independent meaning here as such, both describe independently propagated activity since their PDEs are not coupled. Hence one can think of 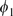 as representing a “full” propagator, which one would encounter in humans, and of 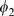 as representing a “depletion” propagator, which then removes activity conduction lacking in lower mammals.

Comparing dispersive and difference distributions for 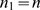 in Tab. 1, we find now that both mean and standard deviation of the difference distribution are larger, but its coefficient of variation is smaller. Thus the difference distribution is sharper. Skewness is indeed more positive for the difference distribution, indicating the increased preference for higher velocities we aimed for. For 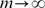 one finds 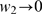, i.e., the difference distribution becomes the dispersive one again. The 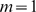 case then also turns out to be least similar to the dispersive one concerning statistical characteristics. Our skewness fit cannot be expected to be faithful outside of the fit range, which however is sufficient for all practical purposes. The only exception is 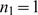 where skewness does not exist, but which may be of interest. The approximation in Eq. (52) extrapolates viably in that case with 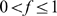, and for simplicity's sake we adopt here the fit for all 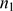. The resulting distribution is shown in Fig. 3B. In comparison to Fig. 1B we see the clear depletion at low velocities for powers 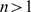, which we aimed to achieve. The extrapolated 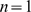 case however does not show a significant depletion. Note that extrapolation of the fit for large powers does not leave the 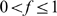 range till 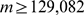. We conjecture that the marginal velocity distribution Eq. (51) is *unimodal* for our choice of 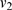 and 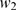. We have checked the 240 cases obtained by varying both 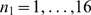 and 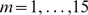. In every case the derivative of 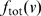 was zero for just one 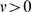. Since 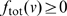, and zero only for 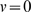 and 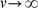, this indicates a single maximum for 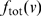.

## Results

### Fits to myelinated fibre diameters in human corpus callosum

How well does the dispersive propagator and its distance-dependent 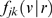 and marginal 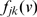 velocity distributions, as well as the difference propagator and distributions derived from it, reflect physiological reality? This is a difficult question to answer since there are surprisingly few studies that have attempted to experimentally quantify the distribution of cortico-cortical conduction velocities in animals or humans. Existing experimental estimates can be divided into two groups: those based directly on conduction latencies, for which the distance-dependent velocity distribution 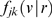 is appropriate, and those based on the transformation of histologically determined axon diameters, to which the marginal velocity distribution 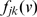 applies. Estimates of cortico-cortical conduction velocities obtained using these approaches cover a wide range, and depend on whether the fibres are myelinated or unmyelinated. For example, myelinated fibres of the corpus callosum are found to have an order of magnitude variation in diameters (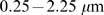 in rat, rabbit, cat and monkey [42]–[45]), with conduction velocities expected to vary roughly linearly with these different calibres. Furthermore, strong regional differences can occur, for example in monkey callosal latency measurements yield a median of 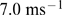 [46], whereas in visual cortex one obtains only 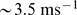 [47]. In the following we will concentrate on fibre diameters and hence the marginal velocity distribution 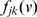, since here some fairly detailed data sets are available. Furthermore, the analysis of latency measurements requires knowledge about the distance between brain areas and adds uncertainties concerning the precise time when transmitted impulses actually lead to a measurable response. However, we will indicate below where latency measurements may solve ambiguities in our fits to data.

For myelinated axonal fibres conduction velocity is found to be linearly related to fibre diameter 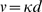. The constant of proportionality 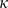 however is not well determined. Below we first concentrate on the work of Aboitiz *et al.* [31], since they provide empirical data for the distribution of callosal axonal diameters in *human* brains. That paper uses 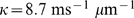. But for example data summarised in Boyd and Kalu [48] suggest that for myelinated axonal fibres with diameter 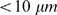 the linear scale factor should be rather 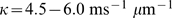. However, we will see below that this uncertainty does *not* influence our data fit directly, but merely scales its result. Aboitiz *et al.* [31] obtained the number of fibres over a given threshold diameter in the corpus callosum of twenty human brains (10 males and 10 females). To this purpose saggitally sectioned and stained post-mortem callosal pieces were examined using light microscopy. In addition electron micrographs were used for one brain. A summary of their data suitable for our purposes is given in Tab. 2. Note that in this table only the last four rows and first two columns contain their directly measured data. The first row and the last two columns are estimates based on other approximate measurements also reported in [31]: On one hand we have subtracted the number of unmyelinated fibres, and on the other hand we have estimated the full unthresholded count. For details see the caption of Tab. 2.

**Table 2:** Threshold counts of myelinated fibres in human corpus callosum based on Aboitiz *et al.* data.

observed threshold diameter 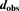	total number of fibres 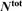	number unmyelinated	number myelinated 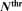
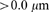	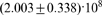	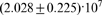	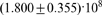
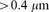	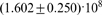	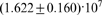	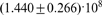
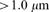	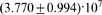	—	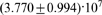
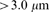	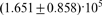	—	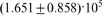
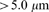	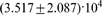	—	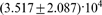

Light microscopy counts (first two columns) are from Tab. III in [31]. The counts for 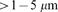 used Loyez stains of only myelinated fibres, but 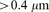 represents Holmes stains, which include unmyelinated fibres. Electron microscopy revealed “about 16%” unmyelinated fibres in the (three segments of the) genu and “usually less than 5%” in the other parts [31]. Using Fig. 1 in [31], we hence estimate the unmyelinated count as 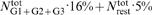, with a 1% error on both percentages. “Approximately 20%” of fibres were not detected with light as compared to electron microscopy [31], hence we estimate the first row from the 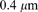 da by dividing by 80% with a 1% error. The first row of the table represents estimates of the average number of fibres in human corpus callosum: total, and distinguished into unmyelinated and myelinated kinds, respectively.

Aboitiz *et al.* [31] counted the number of fibres over a given observed diameter threshold 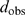. The observed diameters must be corrected for an estimated 65% tissue shrinkage due to formalin fixation and paraffin embedding [31]: 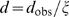 with 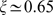. This general shrinkage fortunately maintains the linear relation to conduction velocity: 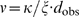. In order to fit this thresholded data, we calculate
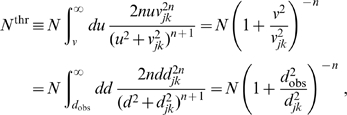
(54)where 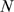 represents the number of all (myelinated) fibres in their corpus callosum sample and 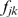 is the marginal velocity distribution Eq. (32) of our newly proposed dispersive propagator. 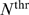 is then the predicted number of fibres having conduction velocities larger than 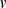. Note that thanks to the linear relationship of diameter to velocity, we can directly compare this to the experimental count 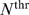 of the number of fibres with a diameter larger than 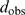. We will then fit the optimal parameters 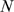 and 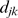, and can relate the latter to the characteristic velocity as 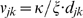. This means that the substantial uncertainties for the velocity scale factor 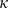 does not directly influence our fit. If 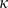 becomes more precisely known the new 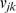 can be obtained simply by multiplication. For reporting velocities we will use the factor 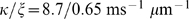 in the following. An effect not covered by the general shrinkage factor 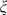 is the possibility of differential shrinkage of the tissue, i.e., fibres of different diameters may have shrunk at different rates in the preparation. Little is known about such effects. Furthermore, fibres typically have a somewhat irregular “oval with dents” cross section in practice, leading to uncertainties in precise determinations of the diameter. Finally, both observer error in the tedious task of counting thousands of fibres and equipment limitations (in particular for small diameters) come into play. For all these reasons it is likely that the 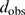 of Tab. 2 should be considered to have some error. In order to take into account all these uncertainties, in particular the unknown differential shrinkage error, we repeat the data fit four times with 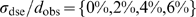.

**Figure 4:**
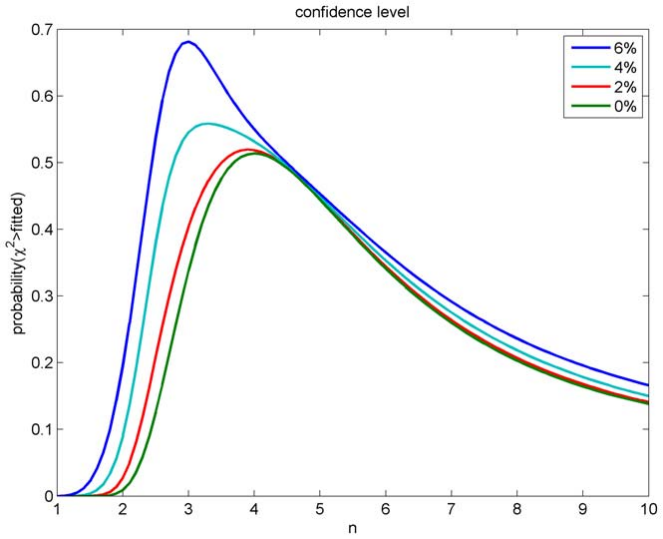
Confidence levels obtained from fits to the data in Tab. 2. The power 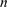 of Eq. (54) was varied in steps of 0.1 for four different uncertainties of the observed threshold diameters 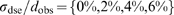. The assumed relative diameter error reflects mainly differential shrinkage. As confidence level the probability that 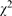 is greater than the fitted 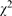 is shown.

In Fig. 4 we show the result of fitting 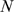 and 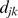 for powers 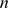 from one to ten. We have repeated the fit in steps of 0.1 in order to obtain smooth curves, but as discussed above only integer powers allow easy computation in terms of PDEs. Shown is the probability of obtaining a 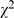 equal to or greater than the actual 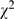, assuming that the data is drawn from the model for a selected 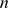 using best-fit parameters. This we will consider as the confidence level of the model with this particular 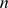. We use here and throughout “generalized chi-square-fitting”, which takes into account errors in both dependent (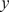) and independent (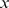) variables at every 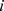-th data point using 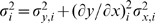, to compare our predicted marginal velocity distributions with the empirically observed data. More advanced approaches, for example those based on Bayesian inference, could in principle give statistically more robust and informative estimates of model parameters. However, the kind of data available to us, from a purely practical point of view, limits the advantages one could obtain with more involved statistical analyses. On one hand, we use here aggregate data from publications, not individual observations, i.e., counts per area and per individual. On the other hand, human data is too scarce and we will see below that the rat data shows systematic deviations from the models. However, our results are sufficiently clear to exclude the long-wavelength velocity distribution for all data, and motivate the use of the dispersive propagator for human and the difference propagator for rat data.

**Figure 5 pcbi-1000653-g005:**
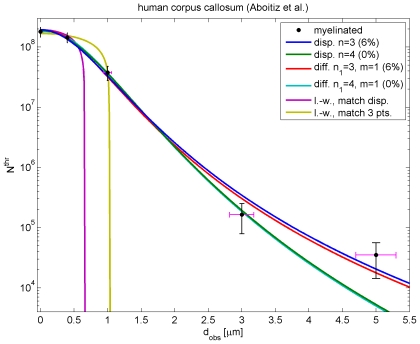
Fits to threshold counts of myelinated fibre diameters in human corpus callosum. The diameter data is collected in Tab. 2, and the fit results with the dispersive Eq. (54) and difference Eq. (55) in Tab. 3. For the dispersive propagator the 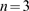 and 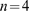 fits are shown, which are optimal assuming 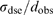 equal to 6% and 0%, respectively. This relative diameter error (magenta error bars: 6%) reflects mainly differential shrinkage. Corresponding difference propagator fits are also shown, which have basically the same confidence levels. Thus these data cannot distinguish the dispersive and difference models, and the former is preferred for its computational simplicity. For the long-wavelength propagator a reasonable fit with Eq. (56) to all data cannot be obtained. Two curves are shown: one matching the median velocity of the dispersive 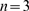 case, the other fitting only the first three data points with 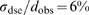.

In Tab. 3 we collect the results for maximum confidence level, i.e., minimum 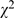. We see that depending on whether the diameter uncertainty is larger or smaller, integer powers 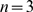 and 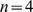 are favoured, respectively. The fitted number of all myelinated fibres 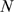 remains well inside one standard deviation of the estimate 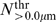 in Tab. 2, but is systematically larger and grows for larger diameter uncertainties. The fitted diameters 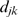 and characteristic velocities 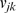 are lower for larger assumed diameter uncertainties. But this mainly reflects the lower fitted powers 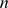, since the extracted mean diameters and velocities remain similar, i.e., larger 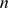 imply “slower” distributions Not surprisingly, larger assumed errors allow better fits, but fit quality is generally satisfactory. Re-fitting with integer 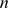 where the best fit is obtained with non-integer 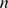 yields similar fit quality with somewhat changed parameters. Given our lack of knowledge concerning the precise diameter uncertainty, it is probably best to consider the 0% case with 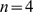, 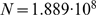, 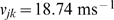 and the 6% case with 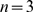, 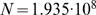, 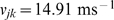 as reasonable limiting cases. They have confidence levels of 51.41% and 68.17%, respectively. The quality of these fits is apparent in Fig. 5. Note that the 0% case predicts 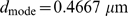 (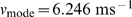) and the 6% case 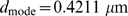 (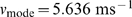). The difference of diameters predicted from these limiting cases is hence likely too small to be detected directly from slice measurements. However, larger 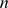 mean overall “slower” diameter distributions. A fit to diameter data naturally reduces the impact of 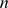 on predicted diameters, but it does so by compensating with an increase of the characteristic 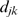. If conduction latencies for large distances are roughly 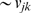, as speculated above, then measuring the resulting larger difference between 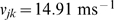 and 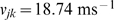 may help distinguishing the 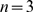 and 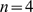 fits experimentally. This nicely demonstrates the (speculative) complementarity of diameter and latency measurements. Conduction latencies for callosal fibres in rhesus monkey gave velocity estimates of median 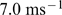 [46]. This may suggest a preference for lower 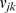, i.e., 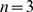 and/or the lower 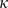 values of Boyd and Kalu [48], if one assumes that the inter-species difference between humans and monkeys is not too drastic. For the 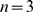 case with 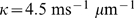, one would find a very similar 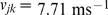 for humans.

**Table 3:** Dispersive and difference fits to threshold counts of myelinated fibre diameters in human corpus callosum.

	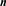	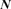	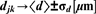	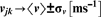	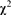	conf.
0%	4	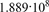	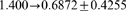	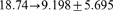	2.292	51.41%
2%	3.9	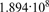	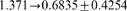	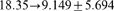	2.262	51.98%
2%	[4]	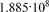	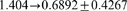	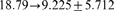	2.266	51.90%
4%	3.3	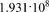	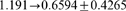	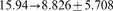	2.067	55.86%
4%	[3]	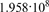	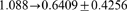	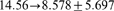	2.131	54.57%
6%	3	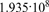	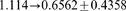	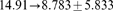	1.502	68.17%
0%	3.8, [ 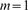 ]	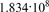	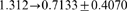	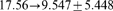	2.118	54.83%
0%	[4], [ 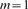 ]	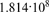	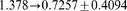	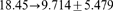	2.153	54.13%
6%	2.9, [ 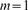 ]	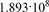	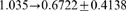	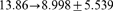	1.525	67.65%
6%	[3], [ 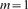 ]	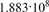	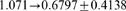	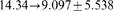	1.534	67.46%

Data of Tab. 2 was fit with dispersive Eq. (54). Fits were repeated assuming uncertainties 0%, 2%, 4%, and 6% of the observed threshold diameters 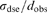 for 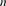 varying from 0 to 10 in steps of 0.1. The values reported here are those of the confidence level peak, i.e., the minimum 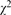, cf. Fig. 4. Where power 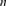 was not an integer at the peak, we also provide the fit with the closest integer 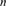, shown in square brackets. For comparison, we repeated this procedure with difference Eq. (55). Difference fits are indicated by a 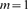 in square brackets, the value we used throughout to minimize the fraction of small diameter fibres, and 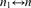, 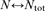, 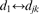, 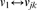 for tabulation. We show results for minimal and maximal diameter uncertainties, again also constrained to integer values. The fit quality of the dispersive and difference fits is basically identical, but difference fits have slightly lower 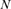 and larger 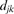. Velocities are calculated here with 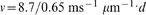.

We have repeated the entire procedure for difference Eq. (51), which yields
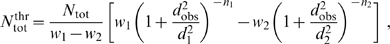
(55)where 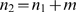 with 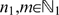. Furthermore, 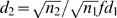 and 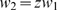 with 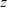 and 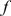 given by Eqns. (44) and (52), respectively. We choose 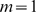 for the difference fits, because for this 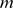 difference and dispersive distributions are most dissimilar, whereas for 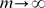 they become the same. We have checked that larger 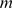 indeed produce results closer to the dispersive fits. Nevertheless, even for 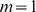 we find confidence levels basically identical with dispersive fits of the same order, see Tab. 3. The difference fits are also shown in Fig. 5, and the similarity to the dispersive curves is evident. The only marginal improvement is that the fitted 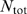 are slightly closer to the experimental value for 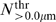 of Tab. 2. Thus our current data for humans is too scarce and imprecise to warrant the introduction of the more complicated difference model, which requires twice the computational effort. A useful fit of the data in Tab. 2 using the long-wavelength Eq. (36)
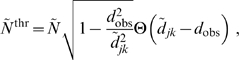
(56)cannot be obtained, since its shape is too much at odds with what is required by the data. It is hence also not clear how to best compare results for the dispersive and the long-wavelength propagator, respectively, since their velocity distributions are so different. One possible suggestion is to match their median velocities, in which case for 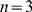 one obtains 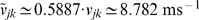, while keeping 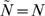. Another possibility is to simply ignore the two data points at largest threshold diameters, which constrain the overall shape, and fit only the first three. Then a fit for 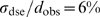 with a probability of 85.43% for 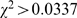 can be obtained. Parameters are 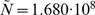 and 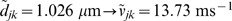. Both of these possibilities are also shown in Fig. 5. We can see that the the long-wavelength propagator matches at low threshold diameters either the new propagator or the first three data points, according to our choice. For comparable numerical simulations one should also adjust the connectivity decay length according to Eq. (25), e.g., 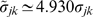 for 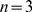. While we strongly recommend using our new propagators, the long-wavelength one perhaps remains attractive for its computational simplicity. But in future one should then use such appropriately “matched parameter values” for 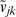 and 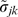. It is interesting that the exponential propagation decay time of Eq. (6) and Eq. (11) with 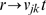 turns out to differ substantially: 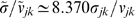 for 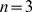. This suggests that the dispersive propagator acts more locally than the long-wavelength propagator.

### Fits to fibre diameters in rat subcortical white matter

**Figure 6:**
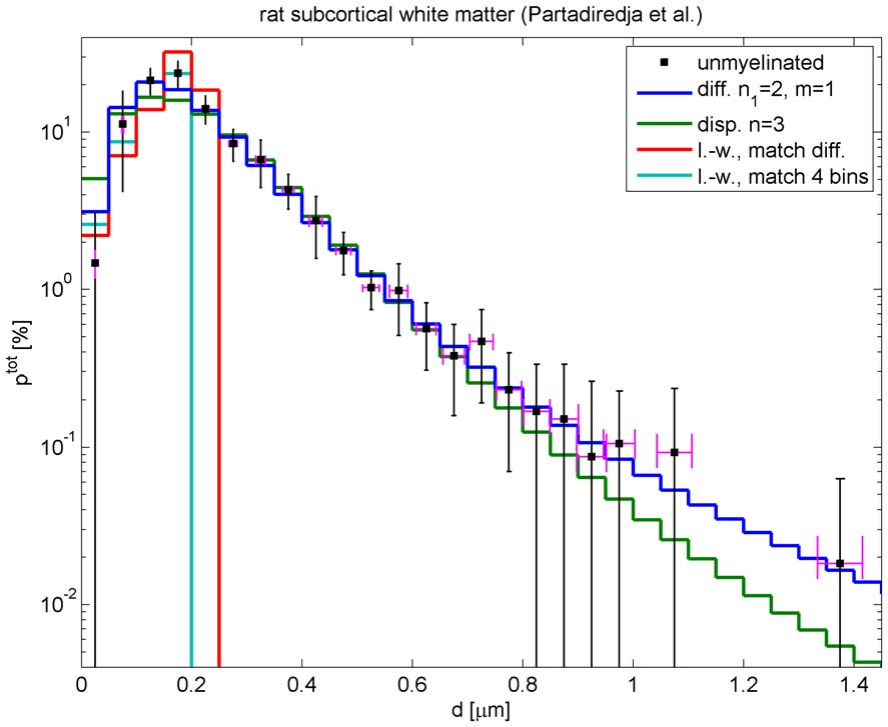
Fits to binned counts of unmyelinated fibre diameters in rat subcortical white matter. The binned diameter data are averages over the unmyelinated data shown in Figs. 4–6 of Partadiredja et al. [36]. 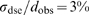 (magenta error bars) has been assumed to reflect mostly differential shrinkage, but fit dependence on this is mild. Fit results using the difference Eq. (57), and its dispersive counterpart Eq. (58), are collected in Tab. 4. For unmyelinated axons the optimal fit with 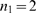, 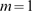 is shown. For comparison, the optimal 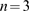 fit with the dispersive propagator is also displayed. It is viable, but has a three times larger 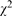. For the long-wavelength propagator a reasonable fit with Eq. (59) to all data cannot be obtained. Two curves are shown for illustration: one matching the median velocity of the difference 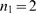, 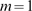 case, the other fitting only the first four data points.

We now turn to animals, where more comprehensive data is available. Partadiredja et al. [36] have recently provided extensive data on axon diameter distributions in rat. As mentioned above, there appears to be a general trend in lower mammals that less of the small diameter fibres are myelinated. Rat data hence provides a convenient test for our difference propagator constructed to deal with such depletion, since we would expect more small diameter fibres and hence easier fits for other animals closer to humans, for example macaques. Furthermore, unlike the human data used above and other data sets from animals, Ref. [36] resolves diameters very finely and hence allows us to pinpoint the strengths and weaknesses of our ansatz. Partadiredja et al. [36] provide their fibre count data in terms of the total densities 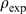 of axons per 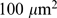 and corresponding percentage histograms 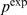 dependent on fibre diameters, see their Tabs. 1 and 2 and Figs. 4, 5 and 6. They provide electromicroscopic results averaged over six adult male Wistar rats, but differentiated according to myelinated and unmyelinated axons of frontal, parietal, and occipital subcortical white matter from both left and right hemispheres.

Partadiredja et al. [36] found differences between left and right hemispheres only for parietal unmyelinated fibres with appreciable statistical significance (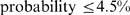). Furthermore, an independent check with a second set of data, albeit at lower magnification, did not confirm even this difference. Thus it is reasonable to average their data for left and right hemispheres. However, it remains difficult to estimate appropriately the errors on their 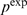 bins by only comparing data from left and right hemispheres. Considering measured mean calibres, they found only one significant regional difference (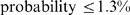, parietal vs. occipital) for unmyelinated axons and one marginally significant one (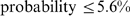, frontal vs. occipital) for myelinated axons. Hence we will proceed here by averaging bin-wise over the six 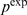 histograms (left and right for frontal, parietal and occipital) available each for myelinated and unmyelinated axons, and simply use the corresponding unbiased estimator of the standard deviation in our fits. It is possible that regional differences could be described with a more sophisticated procedure, but this is sufficient for a parsimonious theoretical description and judging the suitability of our ansatz. We will always take direct averages of the percentage histograms instead of using weighted sums. Considering for simplicity only the average over left and right hemispheres, we thus use 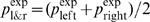 instead of introducing total density weights 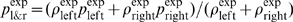. This minimizes the problem of correlated errors, since the errors on 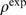 were extracted from the same data as the 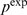 histograms. To predict densities for different diameters one should multiply the averaged 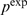 with the likewise averaged 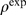.

We relate the marginal velocity distribution Eq. (51) once more linearly 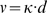 to diameters (the diameters in [36] are already corrected for shrinkage), and obtain for a distribution in bins 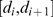 with 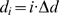 and 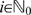:
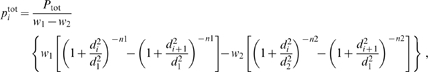
(57)where 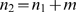 with 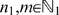. Furthermore, 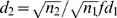 and 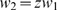 with 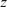 and 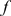 given by Eqns. (44) and (52), respectively. Below we wish to compare the quality of dispersive and difference fits to rat data. The equivalent formula for the dispersive propagator from Eq. (32) is
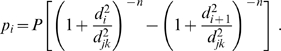
(58)


It is also easy to obtain the corresponding result for the long-wavelength approximation from Eq. (36)
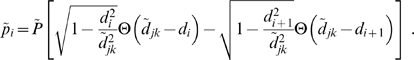
(59)


However, we will see that since 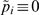 for 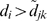, no reasonable fit can be obtained. As in the human case, the complete lack of a large diameter (high velocity) tail in the long-wavelength distribution is at odds with experimental data from rat. The following discussion of the 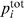 and 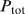 in Eq. (57) applies likewise to the equivalent quantities in Eqns. (58) and (59): If the fitted probability norm 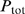 deviates from 100%, then this indicates that the theory prefers a different total density of axons than the experimental mean, namely 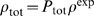. For comparisons with the experiment we further re-norm Eq. (57) by multiplying the predicted 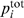 with a factor 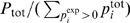. Then the sum of the *predicted* 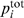 over only those bins where the *experimental* data 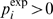 already yields 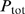. This adjusts for the systematic mismatch between the experimental data, which assigns 100% to the total as sum over those bins which have non-zero empirical entries, and the model, which assigns 100% to the total as sum over all predicted bin counts. Thus we can now truly expect 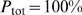 from the fit.

While the linear relation 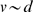 is widely accepted for myelinated axons [49], it is currently not clear how conduction velocity is related to diameter in unmyelinated axons. Theoretical results [50]–[52] tend to favour a 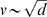 dependence. Experimentally one has found varying results, from squid 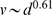 over crab 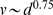 to mammalian C fibres 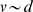, see for example [49] and references therein. Studies of sensory neurons in cat have also suggested a linear relationship [53]. Since currently the situation is inconclusive, we use a linear relationship also for unmyelinated axons, but naturally with a lower 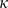 than for myelinated ones. As mentioned, this allows us to fit diameters directly and scale the result to velocities. If we assumed for example 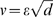 instead, then we would have to relate distributions nonlinearly 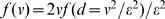. This would inconveniently turn diameter bins of the same size into different size velocity bins. Nevertheless, it is important to note that sublinear diameter powers sharpen up the velocity distribution as compared to the linear case. This is in general detrimental for the quality of our fits. Thus the linear fits to unmyelinated fibre data we provide below need be considered as the ‘best case scenario’. In order to compare better with the previous fits to human data, we again use 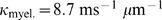 and follow Tab. IV in Aboitiz *et al.* [31] as well in setting 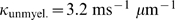, which is based on callosal rabbit data in Ref. [54]. We note once more that for the linear case different assumed 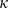 simply re-scale our fit results given below.

Fits of our difference propagator Eq. (57) to the data by Partadiredja et al. [36] are collected in Tab. 4, and compared there with corresponding dispersive fits using Eq. (58). The results for unmyelinated fibres are displayed in Fig. 6. The fit to unmyelinated fibre diameters has a proper optimum concerning the difference propagator model, i.e., upon trying 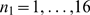 and 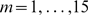 we find an optimal fit for 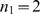 and 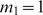 (and thus 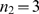). We see that this fit is excellent with a confidence level of 99.997%, likely indicating an overestimate of the errors. Keep in mind though that this is the ‘best case scenario’ linear fit, with lower powers in the relation between velocity and diameter fit quality would deteriorate. Furthermore, it is noteworthy that 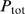 is close to 100% and that the mean diameter of the distribution corresponds very closely to the one estimated from experimental data. This further confirms that the fit performs well. However, and perhaps not surprisingly, the unmyelinated diameters can also be fit with the dispersive propagator, as shown in Tab. 4 and Fig. 6. Once more we find a proper optimum, although for 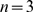. All criteria for a very good fit remain: the confidence level remains high at 91.74%, 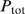 is close to 100% and the mean diameter is close to the experimental value. However, we see that 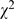 has actually gone up by a factor of *three* as compared to the difference propagator fit. Due to the much higher 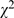, the quality of the dispersive fit is considerably more sensitive to the uncertainty in the relation of velocity to diameter. Inspection of the fit curves in Fig. 6 also suggests that the dispersive fit has a trend of being too wide.

**Table 4:** Difference and dispersive fits to bin counts of fibres diameters in rat subcortical white matter.

	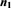	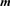	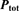	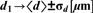	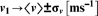	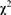	conf.
M	[4]	1	72.33%	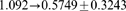	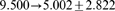	44.15	19.52%
M	[6]	1	74.48%	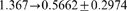	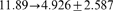	38.23	41.34%
M	[7]	1	75.03%	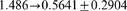	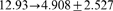	36.69	48.34%
M	[8]	1	75.41%	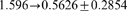	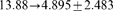	35.60	53.45%
M	[4]	—	58.76%	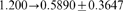	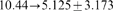	78.84	0.007419%
M	[6]	—	60.89%	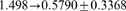	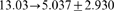	73.32	0.03474%
M	[7]	—	61.44%	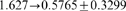	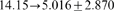	71.88	0.05199%
M	[8]	—	61.83%	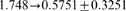	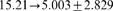	70.85	0.06733%
M	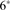	1	85.72%	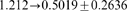	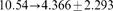	(13.46)	(99.90%)
						60.79	0.08155%
M	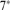	—	84.93%	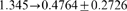	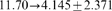	(16.29)	(99.34%)
						141.3	0%
U	2	1	98.92%	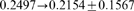	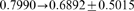	3.861	99.997%
U	3	—	92.96%	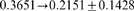	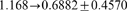	11.96	91.74%

Myelinated (M) and unmyelinated (U) diameter data from the histograms in Figs. 4, 5 and 6 of Partadiredja et al. [36] were fit with difference Eq. (57), and its dispersive counterpart Eq. (58). Dispersive fits are indicated by a missing 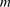 value, and 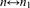, 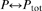, 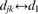, 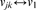 for tabulation. 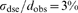 was used for all fits, but dependence on this was mild. For myelinated fibres the difference fit had no optimal 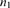, hence several orders were tried as indicated by 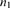 in square brackets. However, 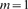 was optimal for any chosen 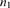. The same holds true for the dispersive fit, and matching 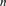 were tried. The entries marked with a 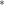 show fits made to diameters 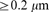 only, i.e., without the first four (myelinated) data bins. Then optimal fit orders exist as shown. Here two sets of 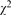 and confidence level are given: in brackets for the large diameters, without brackets compared to the full data. Unmyelinated data directly leads to the shown fits with optimal fit orders. 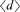 and 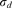 are compatible with the corresponding mean over values in Tabs. 4 and 5 of [36]: 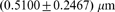 for myelinated and 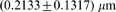 for unmyelinated fibres. Velocities are calculated here with 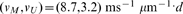.

The large diameter tail in the data, which precludes any direct fit with Eq. (59), is obvious in Fig. 6. One can try once more to match the median velocities of the long-wavelength approximation to that of a more viable fit. From the 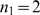, 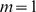 difference fit one obtains 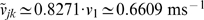. For a fair comparison, we adjust the long-wavelength data norm 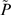 optimally for the number of bins where 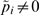. Since 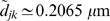, this includes the first five bins and yields 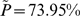. Alternatively one can ignore again the data points at large threshold diameters. The best fit is possible for the inclusion of the first four bins, where 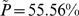 and 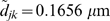 (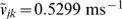), for 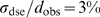 with a confidence level of 72.52% for 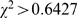. The confidence level for including the first three bins would be 51.26%, and for the first five bins 15.64%. Both the long-wavelength prediction matched in median velocity and the one fit to the first four bins are displayed in Fig. 6. Note that the long-wavelength 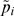 basically rises monotonically with diameter and then suddenly drops to zero. The only slight complication arises for the last non-zero 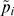, which can rise or fall as compared to the previous bin at smaller diameters. Yet an extended large diameter tail as seen in the data is impossible to achieve.

**Figure 7:**
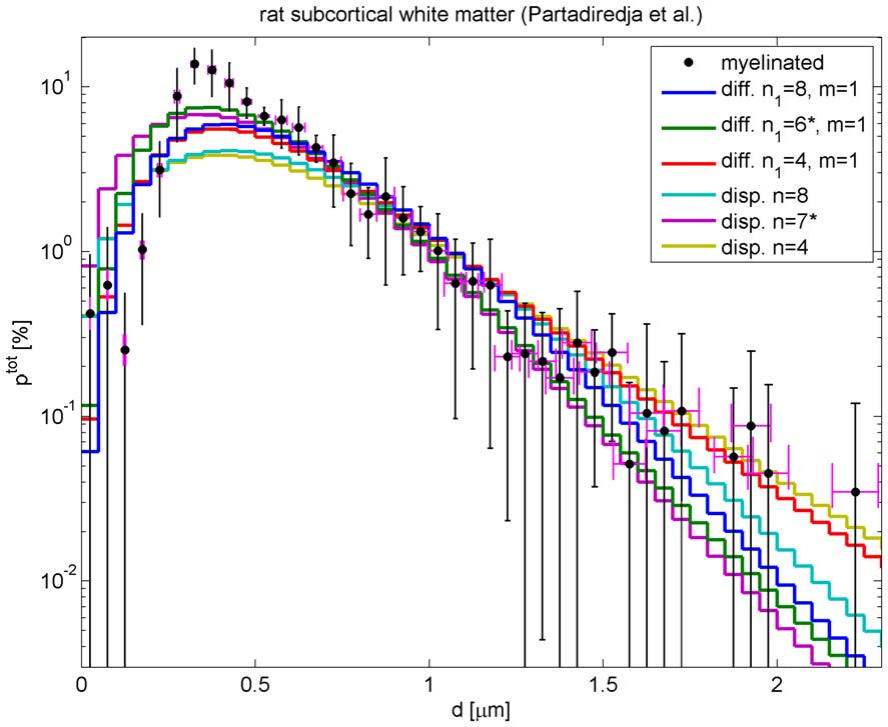
Fits to binned counts of myelinated fibre diameters in rat subcortical white matter. Data and fits are obtained as for Fig. 6, but using the myelinated counts. Two regular difference fit curves are shown: 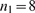 and 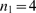, with 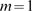 in both cases. Systematic deviations from data around 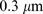 are obvious, but fit quality remains tolerable with a confidence level of 53.45% for 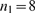. Even larger 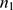 can increase the confidence level to about 70%. For comparison, dispersive fits with orders 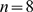 and 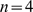 are also shown. Their 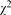 is almost a factor two larger, rendering their confidence level negligible. Fits with the long-wavelength propagator are not show, but fail drastically, cf. Fig. 6. The curves marked with a 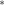 show additional fits for diameters 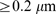 only, i.e., without the first four data bins. Then one can find optimal fit orders for both propagators. These fits are of comparable, excellent quality compared to the reduced data set. But both predict too many small diameter fibres, and hence have negligible confidence levels compared to the full data set, with the dispersive 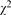 again being about two times larger.

Turning now to our fit results for myelinated fibres, see Tab. 4, we find that the difference propagator has some trouble matching the data. In Fig. 7 this is illustrated by two curves for 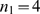 and 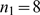, respectively, with 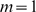 in both cases. Essentially, the experimental distribution is more sharply peaked around 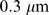 than the difference propagator can easily accommodate. Since the difference propagator becomes more sharply peaked for larger 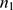, higher powers always provide a better fit in the tested range 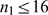, i.e., we cannot find an optimal 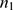 for the model. However, for any given 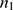 one finds that 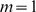 is optimal, since that maximizes the skewness of the distribution. That 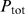 is only about 75% also indicates that our fit has trouble matching the sharp maximum. That said, formally one finds reasonable confidence levels for higher powers of 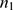, e.g., 53.45% for 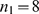. Furthermore, the mean diameters of the distributions are well compatible with the experimental value. While acknowledging the difficulties, we hence conclude that the difference propagator is sufficient for a rough fit even to rat data. Fitting the long-wavelength propagator to these data is of course hopeless, due to the extended large diameter tail. Since we have already considered artificial matching procedures in the unmyelinated case, we do not discuss any long-wavelength fits here. The dispersive propagator also prefers large 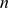 without proper optimum. But if we fix 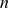 to the same value as the 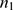 of the difference propagator, then we find roughly a two times larger 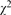 in the dispersive case. This then implies negligible confidence levels for the fit, i.e., the dispersive propagator can be considered as excluded for the myelinated rat data. We show in Fig. 7 two corresponding dispersive curves with 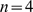 and 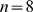, respectively. It is obvious that compared to their difference counterparts they are primarily less able to accommodate the sharp peak around 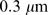.

These results can be summarized also as follows: the depletion of small diameters fibres in the experimental data appears to be even stronger than predicted by the difference model, and excludes the dispersive model. In order to demonstrate that the small diameter data is the culprit, we have repeated the fits, but removed the small diameter bins one by one. We find that after removing the first four bins, and thus for considering only diameters greater than 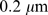, both the difference and the dispersive fit acquire optimal fit orders, namely 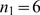, 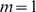 and 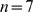, respectively. These fits for larger diameters are also shown in Fig. 7, and are indicated by a 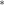 in the legend. As one can see in the figure and in Tab. 4, fit quality is excellent for large diameters for both models. But if one uses the so obtained parameters and compares to the full data set including the small diameter bins, then the confidence levels become negligible. Though again the 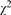 of the dispersive model is about two times larger. It is possible that some experimental problem exists that leads to a systematic underestimate of the number of small diameter myelinated fibres, though we are not in fact aware of any. If that were the case, then the large diameter fits might be closer to reality. Furthermore, the large diameter fit for the difference propagator is actually in accord with the two smallest diameter bins. Hence one could use it instead of say the regular 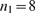 fit, in order to trade a mismatch in the third and fourth bin for an improved description of the peak. Myelinated diameter counts for higher mammals, which show less depletion at small diameters, should be described more easily with the difference propagator. Indeed, this is also suggested by the success of our own fit with the dispersive, and hence “non-depleted”, propagator to human data.

We can now use the fit to unmyelinated rat data to speculate about the human case, for which we have not enough data available for an independent fit. Let us assume that like unmyelinated rat subcortical white matter, also human unmyelinated callosal fibres can be fit with a 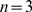 dispersive propagator. Then we can use the two available values from Tab. 2 to determine the characteristic diameter
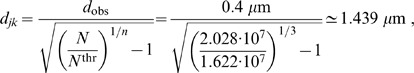
(60)and we thus find 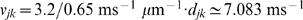. This completes our data fits. We have collected our best fit results in Tab. 5 for easy reference.

**Table 5:** Summary of best propagator fits recommended for use with human and rat data.

label	data set		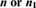	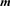	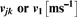	comments
a	human	U	3	—	7.083	speculative, based on 2 data points
b		M	3	—	14.91	optimal fit, 6% diameter uncertainty
c			4	—	18.74	optimal fit, 0% diameter uncertainty
d	rat	U	2	1	0.7990	optimal fit, best 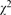
e			3	—	1.168	optimal fit, easier to compute
f		M	8	1	13.88	good overall, best at small diameters
g			6	1	10.54	large diameters only, best at peak
h			4	1	9.500	tolerable overall, easier to compute

Shown is a summary of our best fit results for easy reference. “U” stands for unmyelinated, “M” for myelinated, and difference propagator fits are distinguished from dispersive ones by having an entry for 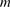. For example, label “c” would indicate choosing a dispersive propagator with parameters 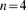 and 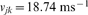 for human myelinated axons.

**Figure 8:**
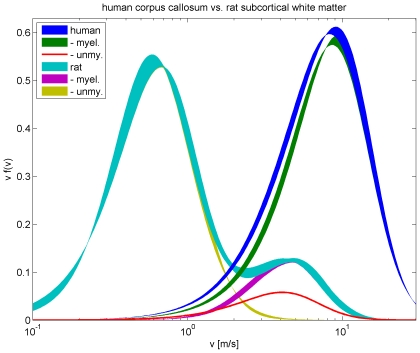
Comparison of combined marginal velocity distributions: human corpus callosum vs. rat subcortical white matter. Shown are unmyelinated and myelinated contributions, and their sum: for human corpus callosum according to Eq. (61) and for rat subcortical white matter according to Eq. (62). The lower and upper borders of the bands are the minimum and maximum envelope, respectively, of all the “best fit” alternatives indicated in these equations, cf. Tab. 5. Since there is only one estimate for the human unmyelinated contribution, in that case a line instead of a band is drawn.

For human callosal fibres, we see from Tab. 2 that 9.19% of fibres are unmyelinated, whereas for rat subcortical white matter we derive from the mean numbers in Tabs. 1 and 2 of [36] that 83.35% of fibres are unmyelinated. We can use these fractions to construct combined marginal velocity distributions in order to understand overall activity conduction properties:
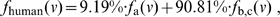
(61)

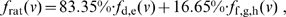
(62)where we have used the labels of Tab. 5 as subscripts to indicate alternatives. In Fig. 8 we show these combined distributions, and the respective myelinated and unmyelinated contributions. To disentangle the curves 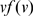 rather than 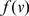 is shown. Thus the area of these curves is normed to mean velocities, rather than to one. Furthermore, to give some feeling for the remaining uncertainty even in our “best fits”, we show bands using the minimum and maximum envelopes of Eqns. (61) and (62). Thus for example the lower border of the “rat – myelinated” band is computed as 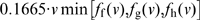. There are of course considerable caveats: the human unmyelinated part is derived speculatively, the rat myelinated part is only a rough fit, and the unmyelinated estimates are in general plagued by the uncertain relation of diameter to velocity. Nevertheless, we expect that the clear differences one can observe here will hold true at least qualitatively: In rat subcortical white matter there are two modes, a dominant, sharp one at low velocities and a broad one at higher velocities. In human corpus callosum one finds only a single, very broad mode at high velocities. One would have to lower the ratio of the myelinated to unmyelinated 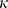 from 8.7/3.2 = 2.7 to about 1.9 to turn the second rat mode into a high velocity shoulder, and further down to about 1.0 to obtain a smooth unimodal distribution. It is biologically implausible to assume that the 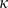 ratio could be so low, since that would abandon the distinction between fibre types. Furthermore, a sublinear relation of velocity to diameter in rat would sharpen the distinction between the modes even more. It is hence likely that rat subcortical white matter operates in two distinguishable velocity regimes, whereas human corpus callosum features only a single one.

### Turing instability analysis

Following Coombes *et al.* [30] we investigate the consequences of the new dispersive propagator in terms of a Turing instability analysis. The Turing instability analysis represents the standard approach to understanding the emergence of spatio-temporal patterns of activity in spatially continuous non-linear dynamical systems [55]–[58]. Specifically it enables the determination of the conditions under which the stability of a homogeneous steady state is lost and the types of patterns of activity that subsequently emerge. This analysis method has been of great utility in understanding self organized pattern formation in a range of physical, chemical and biological systems. In order to facilitate comparison with previously developed long range propagators, we explore the stability of the homogeneous steady state for a Wilson-Cowan or Amari style neural field model in which the mean soma membrane potential is given by
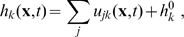
(63)

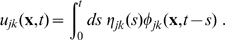
(64)


Here 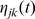 corresponds to the time course of a unitary postsynaptic potential (PSP) and 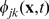 represents the total rate of arrival of presynaptic impulses to neuronal population 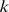 arising from neural population 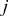. We choose the bi-exponential
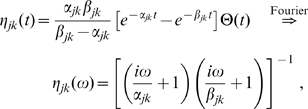
(65)to model PSPs. Therefore the system of equations for the Turing instability analysis are
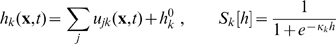
(66)

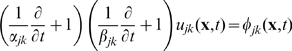
(67)

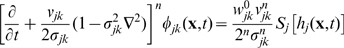
(68)where 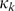 is a gain parameter in the firing rate sigmoid 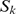. The homogeneous steady state 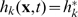 is then given by 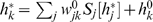. A linearization around this state with 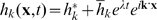 and like perturbations of 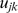 and 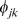 yields the system of equations
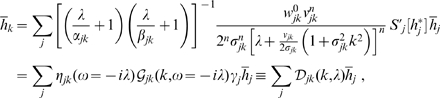
(69)where 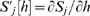 and 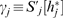. See Refs. [55]–[58] for further detail on this linearization method and its application to the Turing instability analysis.

Nontrivial solutions for 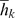 will only exist for 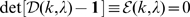, where 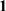 is the identity matrix of appropriate dimension. Solutions to 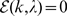 then yield a continuous spectrum of eigenvalues, 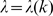, that define the dispersion relationship. Clearly each spatial mode 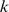 will be stable if the real parts of the corresponding eigenvalues 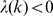, and thus the homogeneous state will be stable to all perturbations if 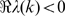 for all 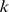. As various model parameters are changed, we expect a critical point will be reached for some 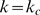 where the real part of the corresponding eigenvalue 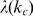 becomes zero. By parametrically moving beyond this critical point the eigenmodes having critical wavenumber 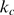 and critical frequency 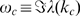 will start to grow, leading to the emergence of spatio-temporal patterns of activity. The expected type of emergent activity can be inferred from the values of 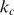 and 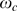. If 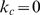 and 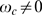 then we expect to see the emergence of spatially uniform periodic oscillations. If 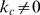 then we expect to see the emergence of spatial patterns of activity that can either be periodic in space but constant in time (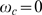), or periodic in space and time (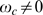). These three bifurcation scenarios are typically referred to as Hopf (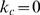, 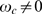), Turing (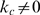, 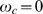), and Turing-Hopf (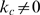, 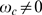) bifurcations, respectively.

**Figure 9:**
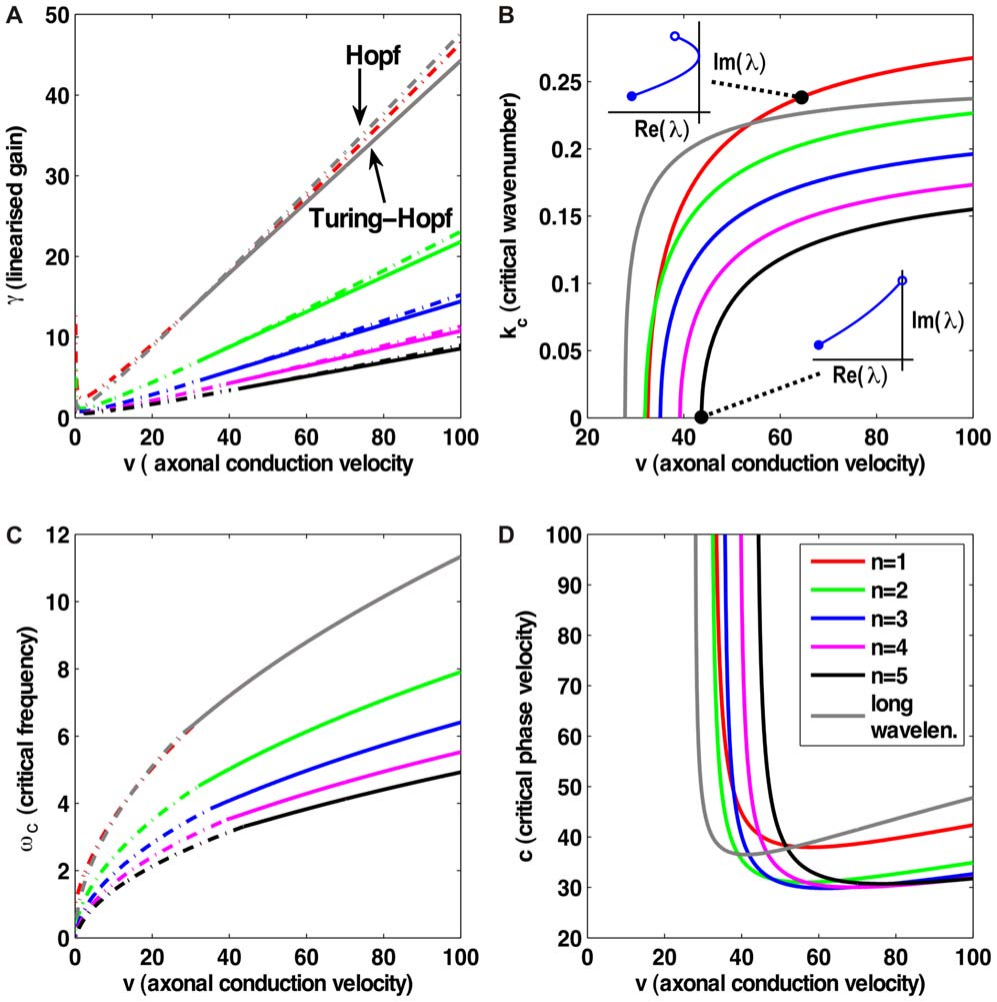
Turing instability analysis of the dispersive and long-wavelength propagators. Bifurcations are investigated by varying the axonal conduction velocity 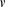 and determining 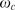, 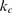, and the critical linearized gain 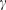. All other model parameters remain at the values discussed in the text. (A) Solid curves represent Turing-Hopf bifurcations (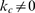), dot-dashed curves Hopf bifurcations (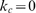). Results for orders 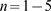 of the dispersive propagator and for the long-wavelength model are shown. Above the Turing-Hopf curves travelling waves emerge, whereas above the Hopf curves bulk oscillations are seen. Stability will be lost at a given 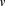 through the less stable bifurcation, which has smaller critical 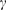. (B) Critical wavenumber 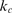 of the Turing-Hopf bifurcation. Insets show the position in the complex plane of the most weakly damped pole under variations of 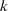 (open circles 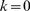, closed circles 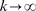) for the dispersive model at the indicated 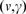. (C) Critical frequency 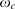 of the less stable bifurcation. (D) Critical phase velocity 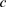, shown where Turing-Hopf is the less stable bifurcation.

For computational purposes it is preferable to split 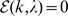 into real and imaginary parts and define 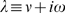:
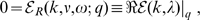
(70)

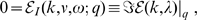
(71)where 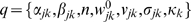 indicates the chosen set of model parameters. Solutions to Eqns. (70) and (71) for a given set of parameters 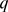 thus yield curves 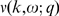 in the 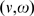-plane parameterised by 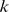, see for example the insets in Fig. 9B. Formally a *Hopf bifurcation* occurs when 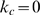 and 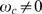 which from Eqns. (70) and (71) gives the condition
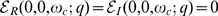
(72)


A *Turing-Hopf bifurcation* occurs when 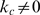 and 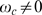 and requires that the solution trajectory 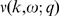 should be a tangent to 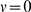 at 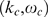, i.e.,
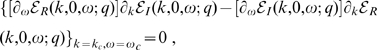
(73)in addition to 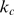 and 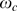 satisfying
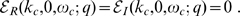
(74)


Eq. (73) can be derived by noting that the total derivatives of Eqns. (70) and (71) with respect to 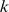 are by the chain rule
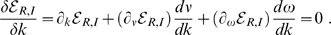
(75)


Solving these two equations by eliminating 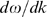 yields
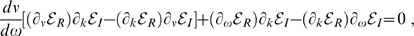
(76)and condition Eq. (73) follows by requiring tangency 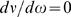. It should be noted that the Turing-Hopf bifurcation results in the emergence of a global pattern with wavenumber 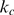 travelling coherently with a critical phase velocity 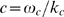. The *Turing bifurcation* occurs when 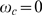 and thus 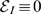 and 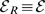, leaving
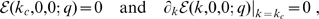
(77)as conditions for the bifurcation. The derivation of Eq. (77) proceeds in a similar manner to that of Eq. (73). In principle one also has to check that such tangential solutions are locally right-bounded, with the local turning point being least stable, but for the dispersive, difference, and long-wavelength propagators under consideration we have found this to be always the case in practice.

In the following, we will investigate the existence of these bifurcations by changing one model parameter 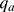 and solving the equations for 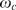, 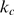, and a second model parameter 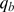. As in Coombes *et al.* [30], for simplicity we consider only two neuronal populations: excitatory (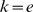) and inhibitory (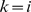). We further simplify the PSP time courses by setting both 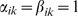 and 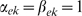. Explicit synaptic delays are not modelled. Because in neocortex excitatory connections have much greater lateral extent than inhibitory connections, we assume that 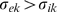, and here set 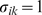 and 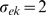. Connectivity weights represent local dominance of inhibition with 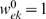 and 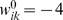, respectively. Uniform axonal conduction velocities 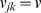 and firing rate functions 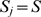 are assumed for simplicity. For subsequent numerical simulations, and without loss of generality, we set 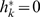 so that the linearized gain 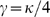. Fig. 9 shows the results of the Turing instability analysis for the dispersive and long wavelength propagator models. Figure 9A shows the critical curves in the 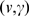 plane. Above each of the respective curves a homogeneous steady state succumbs to dynamical instabilities for 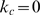 (bulk oscillations, Hopf) and 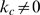 (travelling waves, Turing-Hopf), with the lower curve determining the actual bifurcation at a given 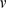. Neither model gives rise to a Turing bifurcation within the admissible parameter space, i.e., Turing bifurcations only occur for negative 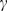. This is in contrast to Steyn-Ross *et al.* [59], in which the effects of *both* chemical *and* electrical (gap junction) synaptic transmission are modelled in a mean field model that includes a long-wavelength propagator. They observed stationary Turing instabilities when homogeneous driving terms similar to 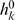 were varied.

Both the dispersive and long wavelength propagators are capable of exhibiting Hopf and Turing-Hopf instabilities. As Fig. 9B shows for the chosen parameter sets, the Turing-Hopf critical wavenumber 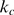 exists only above a finite velocity 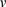 for both dispersive and long-wavelength models. In all cases the 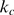 rises smoothly with 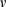 and asymptotes to a maximum value. Considering the critical wavelength 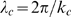 as a typical size, increasing 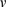 will hence change bulk oscillations first into large travelling waves, which then contract to some minimal size. While the boundaries of instability are of similar shape in the long-wavelength and dispersive propagators, there are nevertheless differences between the loci of the two curves that may be of considerable physiological relevance. As can be seen in Fig. 9A, loss of stability occurs for significantly smaller values of the linearized gain 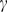 in the dispersive model with 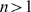 as compared to the long-wavelength one, for a given characteristic conduction velocity 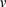. In general this remains true for a reparametrization of the bifurcation curves in terms of the mean, median, or mode of the corresponding velocity distributions, cf. Tab. 1. Because the linearized gain 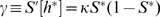 where 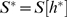 is the steady state firing rate, cf. Eq. (66), changes in 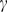 can be achieved by alterations in the steady state firing rate via 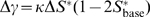. Thus for the 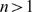 dispersive propagators a smaller change 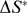 from a given basal firing rate 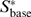 is required to induce pattern formation, as compared to the long-wavelength propagator. This may follow more closely the biological situation, where a range of metabolic and energetic constraints need to be negotiated.

In Fig. 9C we show the critical frequency 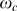 only of the less stable bifurcation, which actually determines the instability. The 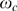 of the propagators are seen to transit smoothly from Hopf to Turing-Hopf with increasing velocity. However, the long-wavelength 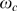 increases more quickly with velocity than the dispersive ones, except for the 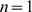 case which is a close match. Thus at a given velocity, 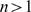 dispersive travelling patterns will emerge at lower critical frequencies than long-wavelength ones. Fig. 9D displays the critical phase velocity 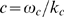 of the emerging patterns. We find a lower 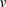 limit for which 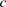 formally diverges, since 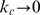 in this limit. Both the dispersive and the long-wavelength critical phase velocities then rise mildly for larger velocities. It is known that developmental changes to the diameters and myelination of axonal fibres occur and partly depend on activity feedback, see for example [60] and references therein. Although highly speculative, it is conceivable that the transitions between bulk oscillations and travelling waves in response to changing conduction velocities, which we have just discussed, could provide a relevant feedback mechanism. That the phase velocity 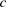 remains close to independent of 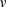 above a threshold – particularly so for larger 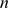 dispersive propagators, less so for smaller 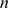 and the long-wavelength case – may then be significant for connectivity development. Obviously significant differences exist between the dispersive and long-wavelength models, especially for larger 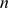. Such differences likely also occur in the bifurcation structure of other parameter planes. The biological implications of these dynamical differences require future detailed investigations, which need to go beyond our qualitative considerations here by restricting the parameters more specifically to experimentally allowed ranges.

**Figure 10:**
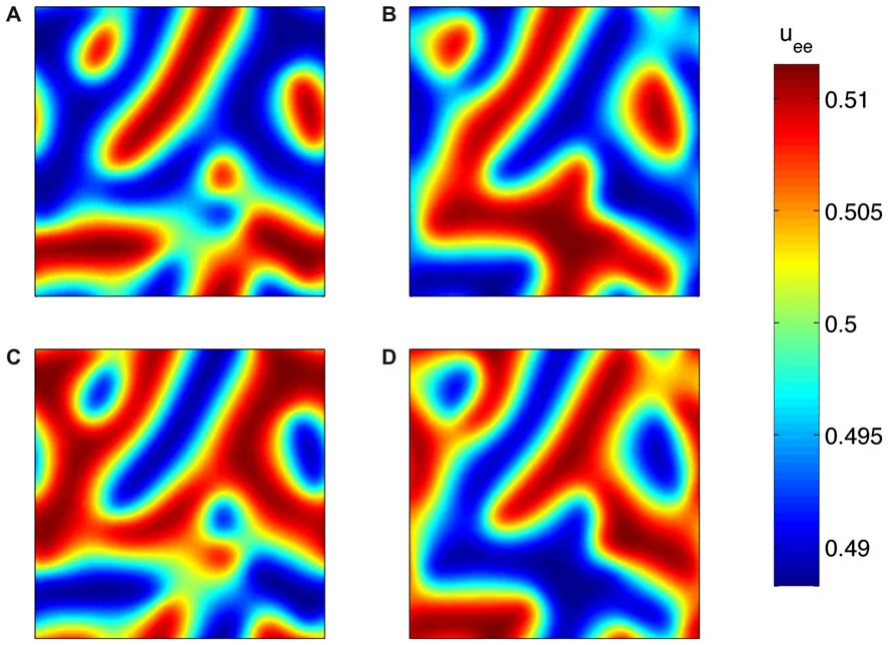
Typical simulation result of the dispersive neural system far beyond a critical Turing-Hopf boundary. Subplots (A)–(D) represent successive snapshots of the spatial patterns of activity in 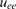 spaced a quarter of the average temporal oscillation period apart. The dispersive propagator model of Eqns. (66)–(68) was computed for 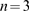 and 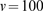 with 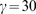 chosen well beyond the Turing-Hopf critical value, cf. Fig. 9A. Spatial derivatives were approximated using finite differences on a regular square grid of 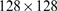 with spacing 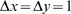. The resulting system of equations was rewritten as a first-order system and integrated using ode45 in MATLAB starting from random initial conditions in 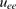. See also the supplementary Video S1 for the corresponding animation.

In order to test the predictions of our linear stability analysis we have performed numerical simulations of Eqns. (66) to (68) over suitably chosen domains. Tab. 6 shows the results of comparisons between the spatio-temporal properties of the numerical simulations for 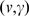 just beyond the Turing-Hopf bifurcation, and the corresponding linear predictions. As can be seen there is excellent agreement for a range of parameters and dispersive propagator orders. In all cases parallel moving stripes were seen beyond the Turing-Hopf bifurcation when integrations were continued for a long enough time (results not shown), but a range of other patterns also occurred depending on the initial conditions. Thus this system likely possesses multiple attractors. By moving further away from the Turing-Hopf bifurcation boundary more complicated, and arguably biologically more plausible, self-organizing behaviour is seen. One such example is shown in Figure 10, see also the corresponding supplementary animation S1. No attempt was made to determine whether the Turing-Hopf bifurcations were subcritical or supercritical in character, though in principle this could have been established by brute force numerical simulation or more elegantly using the method of *harmonic balance* [61].

**Table 6:** Comparison of linear Turing instability analysis with numerical integrations for the dispersive propagator.

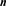	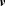	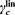 & 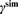	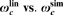	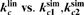	
1	50	22.33	8.04	0.21	linearization
		23	8.38	0.14, 0.22	simulation
	100	44.27	11.34	0.27	linearization
		45	11.73	0.22, 0.28	simulation
3	50	7.23	4.54	0.15	linearization
		8	4.82	0.09, 0.17	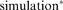
	100	14.43	6.41	0.20	linearization
		14.5	6.92	0.18, 0.26	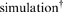

For selected orders 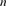 and conduction velocities 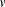 linear Turing instability analyses of Eqns. (66)–(68) were used to predict the critical Turing-Hopf 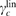, 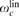, and 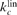, cf. Fig. 9. For numerical simulations, a 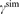 somewhat larger than 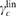 was chosen. The space-averaged 1D temporal Fourier spectrum 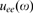 was used to estimate 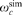 as the maximum of 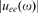. The time-averaged 2D spatial Fourier transform 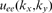 was used to obtain two estimates: 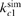 as the 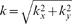 for which 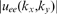 is maximal; and 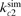 as the 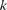 for which the mean of 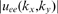 over a circle around the origin with radius 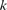 is maximal. For the estimates grid time series of 50 time units with 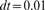 (500 samples total) were recorded, after initial “transients” of 100 (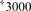) time units were discarded. The spatial grid was 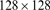 (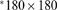) with discretization steps 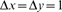.

Performing a Turing instability analysis for either of the difference propagators of Eq. (62) in the 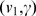 parameter plane revealed a qualitative match with the corresponding 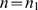 dispersive propagator. In particular, root-loci parameterised with respect to 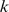, see insets of Fig. 9B for representative examples, reveal that the effect of depleting low velocity fibres in accord with the rat data is to alter the most weakly damped branch of the dispersion relationship only for large values of 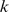, with the low critical wavenumbers observed for the dispersive propagator remaining essentially unchanged. Since the critical curves for the difference propagator would basically reproduce those of the dispersive propagator in Fig. 9, we do not show our additional results for the difference propagator. These predictions also have been verified by numerically integrating Eqns. (66) and (67) using either the un-myelinated or myelinated difference propagator of Eq. (62). Thus on the basis of a linear instability analysis in the context of our current, highly simplified, neural field theory there appear to be no essential dynamical differences between the dispersive and difference propagators concerning bifurcations. However, the dynamical consequences of these propagator forms need to be investigated in future with physiologically more realistic theories of brain electrorhythmogenesis, such as those of Liley *et al.* [26] and Robinson *et al.* [25]. Furthermore, the combination of myelinated and unmyelinated fibre systems, see Eqns. (62) and (61), as well as Fig. 8, reveals differences that are likely to be significant dynamically. However, in order to make meaningful inter-species comparisons of brain dynamics, on one hand more experimental connectivity data, in particular human, is required and on the other hand local brain activity descriptions by mean field theories will have to be adjusted for different species as well.

## Discussion

Understanding the physiological basis of brain dynamics requires one to account for the activity of distributed populations of cortical neurons. Modelling the details of their ongoing communication will generally form an important component of any theoretical description. Continuum mean field models (MFMs) of neural population activity [1],[2],[4],[23],[25],[26] are a particularly useful theoretical tool for bridging the gap between the macro- to mesoscopic assays associated with non-invasive neuroimaging (e.g., EEG or fMRI BOLD) and our knowledge of the underlying microscopic anatomy, physiology and pharmacology. However, MFMs have faced one particularly significant technical challenge: the biologically plausible, yet computationally tractable, propagation of neuronal activity via long-range (cortico-cortical) connectivity. Most current MFMs have followed the pioneering work by Jirsa and Haken [27], which relied on a number of simplifying assumptions in order to derive a numerically efficient and analytically tractable “long-wavelength” propagation PDE. However, in doing so a substantial degree of biological fidelity has been lost, the most crucial of which involves the distribution of axonal conduction velocities. As we have demonstrated here, these PDE formulations have assumed a sharply peaked velocity distribution with a definite cut-off, a feature which is completely at odds with the available empirical evidence that instead suggests rather broad distributions. On this backdrop we have introduced two new long range propagators, the dispersive propagator and the difference propagator derived from it, which retain all the advantages of a PDE formulation but produce broad velocity distributions in keeping with the experimental measurements.

We have provided an extensive analysis of the mathematical properties of these new propagators, and contrasted them with the commonly used long-wavelength model. Of particular note are the following results: First, we can distinguish between the distance-dependent and the marginal velocity distribution. The former is appropriate for the description of experimental measurements of conduction latencies, the latter can be related to the histological determination of fibre diameters in slices. Second, our new propagators predict that more distant brain areas are generally connected by faster fibres. This could be relevant for isochronicity in the brain, see for example [60]. In contrast, the long-wavelength propagator assumes essentially one conduction velocity irrespective of fibre length. Third, if conduction velocities of fibres indeed depend on distance, then typical velocities as extracted from latency and diameter measurements, respectively, are expected to differ. We are not aware that this effect has been described or systematically studied so far. For our new propagators, we can speculate that measuring activity delays over large distances should results in faster velocity estimates than deriving them from the diameters observed in local slices. It may even become possible to falsify propagator models using the constraints from these different types of data, though it is at present unclear whether the current modelling of brain anatomy is accurate enough to allow such conclusions. Fourth, we have shown that two dispersive propagators can be subtracted from each other such that the resulting difference propagator does not exhibit unphysiological properties. This approach, which we have introduced here to deplete the number of low velocity fibres for our rat data fits, can be generalized to the construction of other, more complicated propagators as need arises.

The empirical relevance of our proposed dispersive propagator was illustrated by fitting the associated marginal axonal velocity distribution to histological measurements of axonal fibre diameters obtained from human corpus callosum by Aboitiz et al. [31], see Fig. 5 and Tab. 3. A similar fit with the long-wavelength model was simply impossible, since its functional form entirely mismatched the data. Thus this fit provides for the first time a realistic description of activity propagation in the human brain in the context of MFMs, though unfortunately only callosal data is available in the human case. In order to obtain data from other subcortical matter we turned to the extensive data of Partadiredja et al. [36] for rat. Here we had to introduce the difference propagator to account for the depletion of low velocity axons relative to human in lower mammals. The difference marginal velocity distributions were then shown to fit reasonably well the empirically derived distributions of rat axonal conduction velocities, see Figs. 6 and Fig. 7, as well as Tab. 4. Some systematic deviations between theory and experiment were however visible, caused by an even stronger low velocity axon depletion in the data as compared to our theory. However, it is known that this depletion is the less severe the phylogenetically higher the animal. Indeed we have described the human callosal data successfully here with the “non-depleted” dispersive propagator. Hence it is likely that rat data is a kind of “worst case”, and human data a kind of “best case”, for our new propagators, and reasonable fits were obtained for both.

The results of these fits further allowed us to speculate that the overall velocity distributions of rat subcortical and human callosal fibres are qualitatively quite distinct, see Fig. 8. In rat subcortical matter one finds two modes: a narrow low velocity one corresponding to unmyelinated fibres and a broad high velocity one corresponding to myelinated fibres. Whereas in human corpus callosum there is a single very broad mode at high velocities supported by both fibre types. Therefore rat data (and possibly other mammalian data) may be misleading for the purposes of MFM parameterisation in the context of understanding the spatio-temporal dynamics of human long-range connectivity. Nevertheless, caution must be exercised regarding the actual fitted parameter values due to limitations in the available data: in all cases only data aggregated across individuals and (sub-)regions was used, and this aggregate data was scarce for human and proved difficult to fit for rat. While the advantage of incorporating a high velocity tail with the dispersive and difference distributions is compelling for all data, and the depletion of low velocity fibres with the difference one is important for data from lower mammals, more robust estimates of the fitted parameters will be essential to obtain greater biologically fidelity in future MFM studies. This will depend on the availability of more and “purer” empirical data, as well as the use of more advanced inferential methods for the parameter estimation. For example, an analysis could be made using the Bayesian inferential framework, whereby prior beliefs about the parameters are updated using the available data. One can then simulate from the resulting posterior distributions of the parameters using Markov Chain Monte Carlo (MCMC) methodology [62]. This would have the advantage of more fully taking into account parameter uncertainty, and would allow direct probability statements to be made about the parameters. It would however introduce additional complications in terms of the model fitting process. The outcome would also be dependent on prior beliefs; in the absence of prior beliefs, one could specify prior distributions that are uninformative, but there are several ways of doing so. The frequentist approach that we have followed here is relatively simple to apply, is objective, and comparison of fitted models is straightforward.

In order to investigate the dynamical consequences of our newly proposed propagators, we have embedded them in a Wilson-Cowan or Amari style neural field formulation of local activity which while somewhat simplistic and abstract, is nevertheless more amenable to analytic treatment than biologically more realistic models such as those of Liley *et al.* [26] and Robinson *et al.* [25]. This also facilitates comparisons with previous works [30] and highlights contributions to the observed dynamics from activity propagation. Turing instability analyses were then used to characterise how spatially homogeneous steady states lose stability, and in particular how the patterns of emergent spatio-temporal activity vary as model parameters are changed. These analytic results are based on a systematic linearization of the model, but were confirmed for several cases with numerical simulations of the full equations. The difference propagator was seen to result in essentially the same bifurcation dynamics as the dispersive propagator, at least in this setting. However, considerable differences in the bifurcation dynamics between the dispersive and the long-wavelength propagator were found. Both models predict a transition for increasing axonal conduction velocities from bulk oscillation to travelling waves as dominant instabilities. But the dispersive propagator more easily transits from a homogeneous stable state to self-sustained spatio-temporal patterns. In particular it is found that pattern formation can be induced for smaller changes in neuronal firing rates with the dispersive propagator compared to the more standard long-wave length propagator for given axonal conduction velocities. The biological implications of these features are at present unclear, though it might be speculated that this represents better the biological situation, where a range of metabolic and energetic constraints need to be negotiated in order to ensure that pattern formation, and thus perception, occurs in optimal circumstances.

However, an important qualification needs to be attached to these results. The emergence of self-sustaining spatiotemporal patterns of activity was predicted and simulated with isotropic and homogeneous connectivity. We did not explore here more realistic synaptic footprints, since their inclusion would have considerably complicated the qualitative picture we wished to paint by requiring mappings to coupled PDEs and multiple cortical patches [30],[37],[38]. Furthermore, for quantitative predictions *independent* connectivity data at the level of detail appropriate for MFMs is still lacking, e.g., what fraction of synapses on a local MFM neuron are associated with input from a specific distant region of cortex is generally not known with adequate precision. However, Jirsa and Kelso [63] have elegantly shown that the stability of spatial patterns can be changed by systematically varying the underlying connection topology. Even relatively simple MFMs can then undergo a series of spatiotemporal bifurcations. Since the stability of spatial patterns could also critically depend on heterogeneous connections, considerable uncertainty remains concerning the effects of conduction velocity distributions which we have reported here. It is essential that further work is performed to systematically assess the effects of conduction velocity distributions together with that of heterogeneous connectivity. In this regard it is fortunate that recent advances in modelling the latter [30],[37],[38] can be straightforwardly combined with our work here by changing the underlying conduction PDEs to our dispersive or difference propagators. Future studies with biologically realistic MFMs need to consider carefully whether changing the propagation model would significantly alter their predictions.

Studying the dynamical consequences of the dispersive propagator with more realistic MFMs of brain activity for example may provide greater insight into the role variations in axonal conduction velocity have in health and disease. For instance it has been hypothesised, on the basis of physiological measurement, that general anaesthetics may alter cognitive function through their effects on axonal conduction velocity [64]. A variety of general anaesthetic agents can cause increases in axonal conduction velocity of 10–20% in the peripheral nerves of human volunteers [65]. It is therefore reasonable to speculate that similar changes will occur in other myelinated axons, such as myelinated cortico-cortical fibres. However, more recent studies involving hippocampal tissue slices have shown no effect of the volatile anaesthetic halothane on the conduction velocities of myelinated Schaffer collaterals [66]. Our newly developed propagator, in the context of a realistic mean field theory of electrocortical activity, may help resolve the role that changes in conduction velocity have in determining anaesthetic action.

Now that our novel propagators allow reasonable fits to experimental data in animal and human, we hope for a surge in theoretical investigations of conduction effects, which in turn should stimulate more targeted experimental measurements. In particular, MFMs can now include realistic activity conduction on an empirical basis in the computationally convenient fashion of a PDE for the first time. Furthermore, we expect that our result that fibre diameters and activity latencies estimate different, complementary, aspects of conduction in the brain to be a general feature of underlying velocity distributions, and hence to be of general interest beyond the specific scope of our current work. Finally, our finding that rat subcortical and human callosal fibre systems differ significantly in their velocity distributions beyond simple scaling, while admittedly speculative and clearly limited due to the comparison of different anatomical regions, is of great significance in terms of the inferences we can make about human brain activity from animal models. In particular more attention must be paid to the possible confounding effects that models parameterised on the basis of animal data have in theoretically characterising and accounting for the propagation of axonal activity in human brains.

## Supporting Information

Video S1 Spatiotemporal patterns of activity produced by a Wilson-Cowan or Amari style neural field model with the dispersive propagator. The video shows a numerical simulation (1000 frames at a resolution of 0.01 time units) of Eqs. (66)–(68) with parameters as described below Eq. (77) on a 128×128 grid. An initialisation transient of 300 time units was discarded. The axonal conduction velocity *v* = 100 and the linearized gain *γ* = 30 were chosen well beyond the Turing-Hopf boundary, cf. Fig. 9A. Snapshots of this numerical simulation are presented in Fig. 10.
(5.82 MB AVI)

## References

[1] Wilson HR, Cowan JD (1972). Excitatory and inhibitory interactions in localized populations of model neuron.. Biophys J.

[2] Wilson HR, Cowan JD (1973). A mathematical theory of the functional dynamics of cortical and thalamic nervous tissue.. Kybernetik.

[3] Lopes da Silva FH, Hoeks A, Smits H, Zetterberg LH (1973). Model of brain rhythmic activity: The alpha-rhythm of the thalamus.. Kybernetik.

[4] Freeman WJ (1975). Mass action in the nervous system: Examination of the neurophysiological basis of adaptive behavior through the EEG.

[5] Lopes da Silva FH, van Rotterdam A, Barts P, van Heusden E, Burr W (1975). Models of neuronal populations: The basic mechanism of rhythmicity.. Prog Brain Res.

[6] Zetterberg LH, Kristiansson L, Mossberg KH (1978). Performance of a model for a local neuron population.. Biol Cybern.

[7] Steyn-Ross ML, Steyn-Ross DA, Sleigh JW, Liley DTJ (1999). Theoretical electroencephalogram stationary spectrum for a white-noise-driven cortex: Evidence for a general anesthetic-induced phase transition.. Phys Rev E.

[8] Liley DTJ, Cadusch PJ, Gray M, Nathan PJ (2003). Drug-induced modification of the system properties associated with spontaneous human electroencephalographic activity.. Phys Rev E.

[9] Bojak I, Liley DTJ (2005). Modeling the effects of anesthesia on the electroencephalogram.. Phys Rev E.

[10] Rowe DL, Robinson PA, Gordon E (2005). Stimulant drug action in attention deficit hyperactivity disorder (ADHD): Inference of neurophysiological mechanisms via quantitative modelling.. Clin Neurophysiol.

[11] Wright JJ (1997). EEG simulation: Variation of spectral envelope, pulse synchrony and ≈40 hz oscillation.. Biol Cybern.

[12] Rennie CJ, Wright JJ, Robinson PA (2000). Mechanisms of cortical electrical activity and emergence of gamma rhythm.. J Theor Biol.

[13] Bojak I, Liley DTJ (2007). Self-organized 40 hz synchronization in a physiological theory of EEG.. Neurocomputing.

[14] Wendling F, Bellanger JJ, Bartolomei F, Chauvel PY (2000). Relevance of nonlinear lumped-parameter models in the analysis of depth-EEG epileptic signals.. Biol Cybern.

[15] Robinson PA, Rennie CJ, Rowe DL (2002). Dynamics of large-scale brain activity in normal arousal states and epileptic seizures.. Phys Rev E.

[16] Lopes da Silva FH, Blanes W, Kalitzin SN, Parra J, Suffczyn'ski P (2003). Dynamical diseases of brain systems: Different routes to epileptic seizures.. IEEE Trans Biomed Eng.

[17] Kramer MA, Kirsch HE, Szeri AJ (2005). Pathological pattern formation and cortical propagation of epileptic seizures.. J R Soc Interface.

[18] Liley DTJ, Bojak I (2005). Understanding the transition to seizure by modeling the epileptiform activity of general anesthetic agents.. J Clin Neurophysiol.

[19] Steyn-Ross DA, Steyn-Ross ML, Sleigh JW, Wilson MT, Gillies IP (2005). The sleep cycle modelled as a cortical phase transition.. J Biol Phys.

[20] Phillips AJK, Robinson PA (2007). A quantitative model of sleep-wake dynamics based on the physiology of the brainstem ascending arousal system.. J Biol Rhythms.

[21] Jansen BH, Rit VG (1995). Electroencephalogram and visual evoked potential generation in a mathematical model of coupled cortical columns.. Biol Cybern.

[22] Rennie CJ, Robinson PA, Wright JJ (2002). Unified neurophysical model of EEG spectra and evoked potentials.. Biol Cybern.

[23] Deco GR, Jirsa VK, Robinson PA, Breakspear M, Friston KJ (2008). The dynamic brain: From spiking neurons to neural masses and cortical fields.. PLoS Comput Biol.

[24] David O, Kiebel SJ, Harrison LM, Mattout J, Kilner JM (2006). Dynamic causal modeling of evoked responses in EEG and MEG.. NeuroImage.

[25] Robinson PA, Rennie CJ, Wright JJ (1997). Propagation and stability of waves of electrical activity in the cerebral cortex.. Phys Rev E.

[26] Liley DTJ, Cadusch PJ, Dafilis MP (2002). A spatially continuous mean field theory of electrocortical activity.. Netw-Comput Neural Syst.

[27] Jirsa VK, Haken H (1996). Field theory of electromagnetic brain activity.. Phys Rev Lett.

[28] Hutt A, Atay FM (2006). Effects of distributed transmission speeds on propagating activity in neural populations.. Phys Rev E.

[29] Hutt A, Atay FM (2007). Spontaneous and evoked activity in extended neural populations with gamma-distributed spatial interactions and transmission delay.. Chaos Solitons Fractals.

[30] Coombes S, Venkov NA, Shiau LJ, Bojak I, Liley DTJ (2007). Modeling electrocortical activity through improved local approximations of integral neural field equations.. Phys Rev E.

[31] Aboitiz F, Scheibel AB, Fisher RS, Zaidel E (1992). Fiber composition of the human corpus callosum.. Brain Res.

[32] Stephan KE, Kamper L, Bozkurt A, Burns GAPC, Young MP (2001). Advanced database methodology for the collation of connectivity data on the macaque brain (CoCoMac).. Phil Trans R Soc B.

[33] Kötter R (2004). Online retrieval, processing, and visualization of primate connectivity data from the CoCoMac database.. Neuroinformatics.

[34] Kötter R, Wanke E (2005). Mapping brains without coordinates.. Phil Trans R Soc B.

[35] Mori S, Wakana S, van Zijl PCM, Nagae-Poetscher LM (2005). MRI atlas of human white matter.

[36] Partadiredja G, Miller RH, Oorschot DE (2003). The number, size, and type of axons in rat subcortical white matter on left and right sides: A stereological, ultrastructural study.. J Neurocytol.

[37] Robinson PA (2006). Patchy propagators, brain dynamics, and the generation of spatially structured gamma oscillations.. Phys Rev E.

[38] Daunizeau J, Kiebel SJ, Friston KJ (2009). Dynamic causal modelling of distributed electromagnetic responses.. NeuroImage.

[39] Liley DTJ, Wright JJ (1994). Intracortical connectivity of pyramidal and stellate cells: Estimates of synaptic densities and coupling symmetry.. Netw-Comput Neural Syst.

[40] Hagmann P, Cammoun L, Gigandet X, Meuli RA, Wedeen VJ (2008). Mapping the structural core of human cerebral cortex.. PLoS Biol.

[41] Friston KJ, Harrison LM, Penny WD (2003). Dynamic causal modelling.. NeuroImage.

[42] Waxman SG, Black JA (1988). Unmyelinated and myelinated axon membrane from rat corpus callosum: Differences in macromolecular structure.. Brain Res.

[43] Waxman SG, Swadlow HA (1976). Ultrastructure of visual callosal axons in the rabbit.. Exp Neurol.

[44] Houzel JC, Milleret C (1999). Visual inter-hemispheric processing: Constraints and potentialities set by axonal morphology.. J Physiol Paris.

[45] LaMantia AS, Rakic PT (1990). Cytological and quantitative characteristics of four cerebral commissures in the rhesus monkey.. J Comp Neurol.

[46] Swadlow HA, Rosene DL, Waxman SG (1978). Characteristics of interhemispheric impulse conduction between prelunate gyri of the rhesus monkey.. Exp Brain Res.

[47] Girard P, Hupé JM, Bullier JH (2001). Feedforward and feedback connections between areas V1 and V2 of the monkey have similar rapid conduction velocities.. J Neurophysiol.

[48] Boyd IA, Kalu KU (1979). Scaling factor relating conduction velocity and diameter for myelinated afferent nerve fibres in the cat hind limb.. J Physiol.

[49] Ritchie JM, Waxman SG, Kocsis JD, Stys PK (1995). Physiology of axons.. The axon: Structure, function and pathophysiology.

[50] Matsumoto G, Tasaki II (1977). A study of conduction velocity in nonmyelinated nerve fibers.. Biophys J.

[51] Muler AL, Markin VS (1977). Electrical properties of anisotropic neuromuscular syncytia. II. Distribution of a flat front of excitation.. Biofizika.

[52] Tasaki II, Matsumoto G (2002). On the cable theory of nerve conduction.. Bull Math Biol.

[53] Lee KH, Chung K, Chung JM, Coggeshall RE (1986). Correlation of cell body size, axon size, and signal conduction velocity for individually labelled dorsal root ganglion cells in the cat.. J Comp Neurol.

[54] Swadlow HA, Waxman SG (1976). Variations in conduction velocity and and excitability following single and multiple impulses of visual callosal axons in the rabbit.. Exp Neurol.

[55] Turing AM (1952). The chemical basis of morphogenesis.. Phil Trans R Soc B.

[56] Cross MC, Hohenberg PC (1993). Pattern formation outside of equilibrium.. Rev Mod Phys.

[57] Kapral R, Showalter K (1994). Chemical waves and patterns: Understanding chemical reactivity.

[58] Murray JD (2003). Mathematical biology II: Spatial models and biomedical applications.

[59] Steyn-Ross ML, Steyn-Ross DA, Wilson MT, Sleigh JW (2009). Modeling brain activation patterns for the default and cognitive states.. NeuroImage.

[60] Kimura F, Itami C (2009). Myelination and isochronicity in neural networks.. Front Neuroanat.

[61] Bressloff PC, de Souza B (1998). Neural pattern formation in networks with dendritic structure.. Physica D.

[62] Gamerman D, Lopes HF (2006). Markov chain Monte Carlo: Stochastic simulation for Bayesian inference, volume 68 of *Texts in Statistical Science Series*.

[63] Jirsa VK, Kelso JAS (2000). Spatiotemporal pattern formation in neural systems with heterogeneous connection topologies.. Phys Rev E.

[64] Swindale NV (2003). Neural synchrony, axonal path lengths and general anesthesia: A hypothesis.. Neuroscientist.

[65] Rosner BS, Clark DL, Beck CM (1971). Inhalational anesthetics and conduction velocity of human peripheral nerve.. Electroencephalogr Clin Neurophysiol.

[66] Mikulec AA, Pittson S, Amagasu SM, Monroe FA, MacIver MB (1998). Halothane depresses action potential conduction in hippocampal axons.. Brain Res.

